# Metagenomic and Culture-Based Analyses of Microbial Communities from Petroleum Reservoirs with High-Salinity Formation Water, and Their Biotechnological Potential

**DOI:** 10.3390/biology12101300

**Published:** 2023-10-02

**Authors:** Vitaly V. Kadnikov, Nikolai V. Ravin, Diyana S. Sokolova, Ekaterina M. Semenova, Salimat K. Bidzhieva, Alexey V. Beletsky, Alexey P. Ershov, Tamara L. Babich, Marat R. Khisametdinov, Andrey V. Mardanov, Tamara N. Nazina

**Affiliations:** 1 Institute of Bioengineering, Research Center of Biotechnology of the Russian Academy of Sciences, 119071 Moscow, Russia; vkadnikov@bk.ru (V.V.K.); nravin@mail.ru (N.V.R.); mortu@yandex.ru (A.V.B.); andrey.mardanov@gmail.com (A.V.M.); 2 Winogradsky Institute of Microbiology, Research Center of Biotechnology of the Russian Academy of Sciences, 119071 Moscow, Russia; sokolovadiyana@gmail.com (D.S.S.); mkatusha82@mail.ru (E.M.S.); salima.bidjieva@gmail.com (S.K.B.); e.alexey.mail@yandex.ru (A.P.E.); microb101@yandex.ru (T.L.B.);; 3 Tatar Scientific Research and Design Institute of Oil “Tatneft”, 423236 Bugulma, Russia; bio@tatnipi.ru

**Keywords:** petroleum reservoirs, microbial diversity, metagenome-assembled genomes, halophiles, sulfidogens, MEOR

## Abstract

**Simple Summary:**

A significant amount of residual oil is located in oil reservoirs with high-salinity formation water levels. Microorganisms are capable of producing a number of oil-displacing metabolites, such as volatile acids, alcohols, biosurfactants, biopolymers, and others. The application of microbial-enhanced oil recovery (MEOR) methods is possible after a detailed study of the microbial community of the oil reservoir. The aim of this study was to elucidate the biodiversity of microorganisms in oil reservoirs of Tatarstan (Russia) with high saline formation water in order to select suitable MEOR technologies for these reservoirs. Using metagenomic and cultural methods, it was shown that fermentative and sulfate-reducing bacteria predominate in reservoirs with high-sulfate formation water, whereas in oil reservoirs with low-sulfate water, in the community of *Methanohalophilus* methanogens predominated. A total of 20 pure bacterial cultures were isolated from oil reservoirs. The isolated fermentative bacteria were able to produce oil-displacing metabolites from sugar-containing and protein substrates. However, fermentation products stimulate the growth of sulfidogenic bacteria that form sulfide, which reduces the quality of oil and causes the corrosion of steel equipment. Thus, when choosing MEOR technology, it is necessary to take into account the possibility of activation of fermentative prokaryotes with simultaneous suppression of the growth of sulfidogens in the oil reservoir.

**Abstract:**

The reserves of light conditional oil in reservoirs with low-salinity formation water are decreasing worldwide, necessitating the extraction of heavy oil from petroleum reservoirs with high-salinity formation water. As the first stage of defining the microbial-enhanced oil recovery (MEOR) strategies for depleted petroleum reservoirs, microbial community composition was studied for petroleum reservoirs with high-salinity formation water located in Tatarstan (Russia) using metagenomic and culture-based approaches. Bacteria of the phyla *Desulfobacterota*, *Halanaerobiaeota*, *Sinergistota*, *Pseudomonadota*, and *Bacillota* were revealed using 16S rRNA-based high-throughput sequencing in halophilic microbial communities. Sulfidogenic bacteria predominated in the studied oil fields. The 75 metagenome-assembled genomes (MAGs) of prokaryotes reconstructed from water samples were assigned to 16 bacterial phyla, including *Desulfobacterota*, *Bacillota*, *Pseudomonadota*, *Thermotogota*, *Actinobacteriota*, *Spirochaetota*, and *Patescibacteria*, and to archaea of the phylum *Halobacteriota* (genus *Methanohalophilus*). Results of metagenomic analyses were supported by the isolation of 20 pure cultures of the genera *Desulfoplanes, Halanaerobium*, *Geotoga*, *Sphaerochaeta*, *Tangfeifania,* and *Bacillus*. The isolated halophilic fermentative bacteria produced oil-displacing metabolites (lower fatty acids, alcohols, and gases) from sugar-containing and proteinaceous substrates, which testify their potential for MEOR. However, organic substrates stimulated the growth of sulfidogenic bacteria, in addition to fermenters. Methods for enhanced oil recovery should therefore be developed, combining the production of oil-displacing compounds with fermentative bacteria and the suppression of sulfidogenesis.

## 1. Introduction

Oil field microorganisms are involved in the transformations of oil, gas, and oil-bearing rock and induce the corrosion of steel equipment, causing significant economic losses [1,2]. Microbial processes of oil biodegradation occur in reservoirs with temperatures below 80 °C [3]. Abundant information has been obtained on microbial diversity and geochemical activity in oil fields with low-saline formation water, characterized by a wide temperature range, using culture-based, radiotracer, and 16S rRNA-based approaches [4,5,6,7]. Metagenome analysis of microbial communities has been carried out for various hydrocarbon resource environments and petroleum reservoirs in Canada [8], China [9,10,11,12,13], USA [14], the North Sea [15], and Brazil [16,17], while metagenomic studies of the microorganisms inhabiting oil reservoirs with high-salinity formation water are scarce [18,19,20,21,22].

The composition of microbial communities depends on such physicochemical conditions of the oil field as temperature, salinity, and the pH of injected and formation water, as well as on sulfate and sulfide concentrations [7,13,23,24,25,26]. The major physiological groups of oil field microorganisms usually comprise fermentative, syntrophic, iron-reducing, sulfate-reducing, and methanogenic prokaryotes [4,27,28,29]. The fractal nature of microbial food chains in oil reservoirs has been hypothesized [30]. 

Oil field development involving water flooding results in the activation of microbial processes of oil biodegradation [31]. The injection of surface water containing dissolved oxygen, nitrate, or organic substrates may result in changes to the physicochemical conditions and shifting of the dominant microbial populations in the near-bottom zone of injection well; its effect, however, decreases with increased distance from the injection zone [26,30,31,32,33]. Radiotracer studies have revealed methanogenesis to be the main terminal process of oil biodegradation in sandstone collectors with sulfate-free formation water, while sulfate reduction is dominant in the carbonate and sandstone collectors with sulfate-containing water [34,35].

The oil reservoirs of Tatarstan have been operational for over 60 years. The preferential recovery of light conditional oil from reservoirs with low-salinity formation water resulted in an increased share of recoverable resources of heavy oil from reservoirs with formation water salinity exceeding that of seawater. Microorganisms are known to produce a range of oil-displacing metabolites, including biosurfactants, biopolymers, fatty acids, solvents, enzymes, and gas [36,37,38,39]. These metabolites and microbial biomass affect the properties of formation water, oil, and oil-bearing rocks, as well as the direction of hydrodynamic flows in the reservoir. 

Trials of various technologies for microbial enhanced oil recovery (MEOR) have previously been carried out at the Tatarstan oil fields [40,41,42]. The possibility of the directional regulation of activity of the reservoir microbial community with the production of oil-displacing metabolites from oil has been shown for sandstone Devonian deposits of the Bondyzhskoe and the Romashkinskoe oil fields, exploited by flooding with a mixture of formation and fresh river water [32,42]. Preliminary investigations of the oil field microorganisms are required for the development of MEOR technologies for oil reservoirs with high-salinity formation water flooded with brines. 

The aim of this study was to determine the composition of microbial communities of oil reservoirs with high-salinity formation water in Tatarstan (Russia) using cultural, 16S rRNA-based, and metagenomic approaches, to compare the metagenomic data of the dominant prokaryotes carrying out the key processes (sulfate reduction, methane formation, and degradation of petroleum hydrocarbons) with the features of isolated pure cultures, and to describe their potential for MEOR or the suppression of corrosion.

In this study, new information was presented on the microbial diversity and biotechnological potential of the microbiota of oil reservoirs with high-saline formation water. The results of geochemical characterization of petroleum reservoirs and the analysis of 75 metagenome-assembled genomes (MAGs) were combined with data from the study of a large collection of isolated difficult-cultured prokaryotes. The formation of oil-displacing metabolites by aerobic and anaerobic isolates was studied. The results of fundamental research allow us to choose the most preferable method of enhancement of oil recovery for oil reservoirs with carbonate collectors and high-saline formation water.

## 2. Materials and Methods

### 2.1. Site Description and Sampling

Two injection (IW) and eight production water (PW) samples were collected from petroleum reservoirs with high-salinity formation water located in Tatarstan (Russia). Oil fields with carbonate oil-bearing collectors (Arkhangelskoe and Romashkinskoe (bed 302)) were located at the depth of 808–998 m below sea level (bsl); oil fields with sandstone reservoirs (Novo-Elhovskoe (PW2706, PW2707, PW7511) and Sabanchinskoe) were located at the depth 1225–1744 m bsl. The temperature of these deposits ranged from 19 to 27 °C. Oil from the Arkhangelskoe oil field with a density 0.924 g·cm^−3^ (20 °C) under surface condition fell into the category of heavy oil, while the density of the oil from other oil fields was essentially lower and ranged from 0.856 to 0.908 g·cm^−3^ (20 °C) [42]. Formation water from bed 302, Romashkinskoe oil field, contained 250–300 mg sulfide per liter [43], which was practically undetected in other studied samples. Characteristics of the oil reservoirs are listed in Table S1. The Sabanchinskoe oil field was exploited with water flooding, with high-salinity production water reinjected (PWRI) into the oil reservoir after oil separation (injection water 1 = IW1). Other studied oil fields were exploited with the injection of a mixture of production water and fresh river water (injection water 2 = IW2). Other samples represented production water collected at Romashkinskoe oil field, bed 302 (PW15500, PW35943), Arkhangelskoe oil field (PW7860), Novo-Elhovskoe oil field (PW2706, PW2707, PW7511), and Sabachinskoe oil field (PW1959, PW1968).

Water samples were collected in June 2021 at the well head of injection and production wells, dispensed into sterile bottles, hermetically sealed without air bubbles, and used for microbiological analyses on the day of sampling. The samples for chemical analyses were stored at 6 °C. Ten water samples for 16S rRNA-based molecular studies (1 L each) and three water samples for the metagenomic analysis of microbial communities (8.5 L each) were fixed with ethanol (15%, *v*/*v*) on the day of sampling and filtered through 0.22-µm membranes (Sartorius, Göttingen, Germany). The filters were then stored at −20 °C prior to analysis. 

### 2.2. Chemical Analyses

For chemical analysis, the water samples were filtered through 0.2 µm filters (Merck Millipore, Darmstadt, Germany) and analyzed with ICP-MS and ion chromatography, as reported previously [44]. Gas composition in the grown cultures was determined using a Kristall-5000.1 gas chromatograph (Chromatec, Yoshkar-Ola, Mari El, Russia) equipped with a katharometer detector. Volatile fatty acids were analyzed using a GC-2010 Plus gas chromatograph (Shimadzu, Kyoto, Japan). The concentration of nitrate ions was determined using an Expert-001 ionometer (Econix-Expert, Moscow, Russia), as described previously [45]. Nitrite was determined using Quantofix test strips (Macherey-Nagel, Düren, Germany). Sulfide was monitored with the colorimetrical method by Trüper and Schlegel [46]. 

### 2.3. DNA Extraction and the 16S rRNA Gene Library Preparation and Sequencing

Microbial biomass from injection and production water samples (1 L each) fixed with ethanol (85:15, *v*/*v*) was collected via filtration through 0.2 µm sterile cellulose nitrate membranes (Sartorius, Göttingen, Germany). The total DNA was extracted from filters using a DNeasy PowerSoil Pro DNA isolation kit (QIAGEN, Hilden, Germany) according to the manufacturer’s instructions. The PCR amplification of 16S rRNA gene fragments comprising the V3–V4 variable regions was carried out using the universal prokaryotic primers 341F_Fr (5′-CCT AYG GGD BGC WSC AG-3′) and 806R_Fr (5′-GGA CTA CNV GGG THT CTA AT-3′) [47]. The PCR fragments were barcoded using the Nextera XT Index Kit v.2 (Illumina, San Diego, CA, USA). All PCR fragments were then mixed and sequenced on Illumina MiSeq (2 × 300 nt from both ends). Pairwise overlapping reads were merged using FLASH v.1.2.11 [48]. The final dataset consisted of 2,198,811 16S rRNA gene reads (Table S2). 

The obtained sequences were binned based on 97% sequence similarity to designate the unique operational taxonomic units (OTUs) using USEARCH [49]. The taxonomic identification of OTUs was performed through searches against the SILVA v.138 rRNA sequence database using the VSEARCH v. 2.14.1 algorithm [50]. To calculate OTU abundances, all reads obtained for a given sample were mapped to OTU sequences at a 97% global identity threshold using USEARCH. 

The Chao1 and Shannon diversity indices at a 97% OTU cut-off level were calculated using USEARCH v.11 [49]. To avoid sequencing depth bias, the numbers of reads generated for each sample were randomly sub-sampled to the size of the smallest set (3756 reads from IW1 sample) using the “otutab_rare” command of USEARCH.

### 2.4. Metagenome Sequencing and MAGs Assembly

The metagenomic DNA samples were sequenced using the Illumina platform (Illumina, San Diego, CA, USA). The TruSeq DNA library was sequenced on an Illumina HiSeq2500 instrument in a paired reads mode (2 × 150 nt). A total of 111, 122, and 100 million read pairs (33, 37, and 30 Gbp) were obtained for samples PW15500, IW1, and IW2, respectively. Adapter sequences and low-quality read ends were trimmed using Cutadapt v.1.17 [51] and Sickle v.1.33 (https://github.com/najoshi/sickle, accessed on 20 August 2022), respectively. Contigs were assembled using the metaSPAdes v.3.15.4 and binned into MAGs using three different programs: MetaBAT v2.15 [52], MaxBin2.0 v2.2.7 [53] and CONCOCT 1.1.0 [54]. The results of the three binning programs were merged into an optimized set of bins using DAS Tool 1.1.4. The completeness of the MAGs and their redundancy (possible contamination) were estimated using the CheckM v.1.1.3 tool [55]. The taxonomic assignment of the obtained MAGs was performed using the Genome Taxonomy Database Toolkit (GTDB-Tk) v.1.5.0 [56] and Genome Taxonomy Database (GTDB) v.202 [57]. 

KEGG gene annotation was carried out using the KAAS web server, and the KEGG modules’ completeness was evaluated using the KEMET Python tool [58]. CCA analysis was calculated in the vegan v2.6-2 R package using the cca function, and the significance of CCA ordination was tested using the ANOVA function in vegan.

All bins were placed into the GTDB reference tree using the GTDB-Tk v1.5.0 program, and the closest reference genome for each bin in the GTDB tree was identified using ape v5.6-2 package in R. ANI and AAI values with closest reference genomes were calculated using scripts from the Enveomics Collection.

### 2.5. Media Composition for the Enumeration and Isolation of Prokaryotes

The numbers of microorganisms of the major physiological groups were determined by inoculating a tenfold dilution of the water samples into liquid media in two replicates. The results were calculated using the McCready tables of the most probable numbers [59]. The basal saline mineral medium (SMM) used for the enumeration of sulfate-reducing, denitrifying, and methanogenic microorganisms contained (per liter distilled water): 45.0 g NaCl, 3.0 g MgCl_2_·6H_2_O, 1.5 g K_2_HPO_4_, 0.75 g KH_2_PO_4_, 0.9 g NH_4_Cl, 0.6 g KCl, and 0.2 g CaCl_2_·2H_2_O. Aerobic organotrophic bacteria (AOB) were enumerated in the SMM medium supplemented with 1.0 g·L^−1^ glucose, 5.0 g·L^−1^ bacto tryptone, and 2.5 g·L^−1^ yeast extract at pH 7.0–7.2. Hydrocarbon-oxidizing bacteria (HOB) were assessed in the SMM medium supplemented with the mixture of C_10_–C_16_ *n*-alkanes (1% *v*/*v*). Air was a gas phase for aerobic bacteria. Sulfate-reducing bacteria (SRB) were assessed using sulfide production in the serial dilutions in the SMM medium amended with 2.8 g·L^−1^ Na_2_SO_4_, 4.0 g·L^−1^ sodium lactate, 0.5 g·L^−1^ yeast extract, 0.5 g·L^−1^ NaHCO_3_, 0.2 g·L^−1^ FeSO_4_, and 0.2 g·L^−1^ Na_2_S·9H_2_O; pH 7.0–7.2. For the enumeration of denitrifying bacteria, the SMM medium was amended with 0.85 g·L^−1^ NaNO_3_ and 2.0 g·L^−1^ sodium acetate. Their growth was monitored through the changes in nitrate and nitrite concentrations in the medium, and of dinitrogen in the gas phase. For fermentative bacteria, the SMM medium was supplemented with 4.0 g peptone, 10.0 g glucose, 0.5 g Mohr’s salt (FeSO_4_·(NH_4_)_2_SO_4_·6H_2_O), and 0.5 g cysteine hydrochloride; pH 6.5–7.0. Argon was the gas phase for the anaerobic bacteria mentioned above. The number of methanogens (MET) was assessed using methane accumulation in a gas phase in SMM medium supplemented with 2.5 g·L^−1^ sodium acetate, 1.5 mL·L^−1^ methanol, 0.5 g·L^−1^ yeast extract, 2.5 g·L^−1^ NaHCO_3_, and 0.5 g·L^−1^ Na_2_S·9H_2_O, pH 7.0, with H_2_/CO_2_ (4:1, *v*/*v*) as the gas phase. A trace elements solution (1 mL·L^−1^) from Pfennig and Lippert [60] and a vitamin solution (1 mL·L^−1^, [61]) were added to the media for anaerobic bacteria. Incubation was carried out for 14 days at 25 °C; the absence of growth was registered after 30 days of incubation. All inoculated test tubes were examined using epifluorescence microscopy under an Axio Imager.D1 microscope (Carl Zeiss, Oberkochen, Germany). 

The aerobic strains were isolated from the aerobic organotrophic enrichments using successive plating on the medium with a similar composition amended with agar (2% *w*/*v*). The anaerobic strains were isolated from the highest dilution of anaerobic enrichments through successive inoculation of the respective liquid media. Strains were incubated at 28–30 °C. The purity of the strains was checked using the microscopy of colonies or cultures grown in liquid media and by sequencing the 16S rRNA genes, as described previously [62]. 

The production of low fatty acids by fermentative enrichments grown on various organic substrates was estimated in the SMM medium with 10% NaCl (*w*/*v*), 0.3 g·L^−1^ yeast extract, and 0.5 g·L^−1^ cysteine hydrochloride, amended with sodium thiosulfate (2.0 g·L^−1^) or without it. Peptone, starch, sucrose, and glucose were added each at concentration at 5 g·L^−1^, and fumarate, lactate, glycerol, and glutamate at 2 g·L^−1^. 

The effect of nitrate on sulfide production by sulfidogenic bacteria was estimated as described previously [25]. The enrichment culture obtained through inoculating the 15500 water sample into the medium with peptone and glucose for fermentative bacteria was used in these experiments. The basal saline mineral medium (SMM) with yeast extract (0.5 g L^−1^), sodium lactate (2 g L^−1^), sucrose (5 g L^−1^), trace elements solution (1 mL·L^−1^) [60], vitamin solution (1 mL·L^−1^) [61], and Na_2_S·9H_2_O (0.05 g L^−1^) was amended with Na_2_SO_4_ (2.8 g L^−1^) or Na_2_S_2_O_3_·5H_2_O (1.6 g L^−1^) for stimulating the growth of sulfate-reducing (SR) or thiosulfate-reducing (TSR) components of the enrichment, respectively. Ca nitrate in concentrations of 0, 0.5, 1.0, 1.5, and 2.0 g L^−1^ (calculated for the nitrate ion) was added to media with sulfate and thiosulfate. Enrichments were incubated at 22 °C for 28 days. Sulfide and nitrite production was monitored in the media in the course of these experiments.

### 2.6. Nucleotide Sequence Accession Number

Nucleotide sequences of the 16S rRNA gene of pure cultures were deposited into GenBank under accession nos. OQ348127, OQ348135, OP459260, OP459261, OP459263, OP459266, OP459271, OP459272, OP459302, OP459305, OP503444, OP503475, OP503477, OP503479, OP588951, OP848502, OP849624, OP849652, and OP849684. The 16S rRNA gene V3–V4 fragment sequences and metagenomic sequences of microbial communities have been deposited in the NCBI Sequence Read Archive (SRA) and are available via the BioProject PRJNA927132.

## 3. Results

### 3.1. Formation Water Chemistry and Abundance of Cultivable Microorganisms

Formation water samples collected from petroleum reservoirs of Tatarstan were characterized by high salinity and belonged to the chlorine–calcium type (Table 1). The production water obtained from reservoirs with carbonate oil-bearing collectors (Arkhangelskoe and bed 302 of the Romashkinskoe) had salinity levels from 62.55 to 116.35 g·L^−1^ and contained high levels of sulfates (0.768 to 6.362 g·L^−1^). Production water sampled from reservoirs with sandstone oil-bearing collectors (Sabanchinskoe and the Pashiiskii horizon of the Novo-Elkhovskoe) had salinity from 97.69 to 157.85 g·L^−1^ and contained a lower level of sulfates (0.006–0.455 g·L^−1^). The studied samples also contained iron (6.2–57.8 mg L^−1^), vanadium (0.03–0.6 mg L^−1^), barium (0.1–385 mg L^−1^), and phosphorus (<0.5–5.4 mg L^−1^) (data not presented). The pH levels in the samples were between 6.11 and 7.55. Acetic acid (up to 11.0 mg L^−1^) was detected, while C_3_–C_5_ low fatty acids were not revealed. 

The examined production water samples and injection water 1 (IW1), represented by recycled formation water, were characterized by very low numbers and a diversity of cultivable microorganisms (Figure S1). However, injection water 2 (IW2), comprising a mixture of fresh river water and production water, was characterized by a higher number of microorganisms. Cultivable aerobic organotrophic bacteria were found in the IW2 sample (10^4^ cells·mL^−1^) and in two PW samples (PW15500 and PW2706; ≤10 cells·mL^−1^). Aerobic hydrocarbon-oxidizing bacteria were not detected. Anaerobic denitrifying/nitrate-reducing bacteria estimated through nitrite and N_2_ production were found in the samples from three production wells. Sulfate-reducing and methanogenic prokaryotes were registered in six PW samples at ≤ 10–10^2^ cells·mL^−1^. Fermentative prokaryotes were the most abundant cultivable group obtained in all studied water samples (10–10^7^ cells·mL^−1^).

### 3.2. Microbial Community Composition Based on 16S rRNA Gene Metabarcoding

The sequencing of the 16S rRNA gene amplicons from two IW samples and eight samples of PW resulted in a total of 76,787 sequences of variable V3–V4 fragments of the 16S rRNA genes. The clusterization of the sequences with 97% similarity resulted in 47 to 410 OTUs per sample (Table S2). According to the Chao1 and Shannon alpha-diversity indices, the highest diversity of microbial communities was observed in IW2 (a mixture of production water and fresh river water) and in PW2707. In other samples, the values for the indices were over two times lower, 43.1–189.7 and 0.678–1.17, respectively, which was in agreement with the low diversity of microbial communities revealed in the oil reservoirs. 

**Figure 1:**
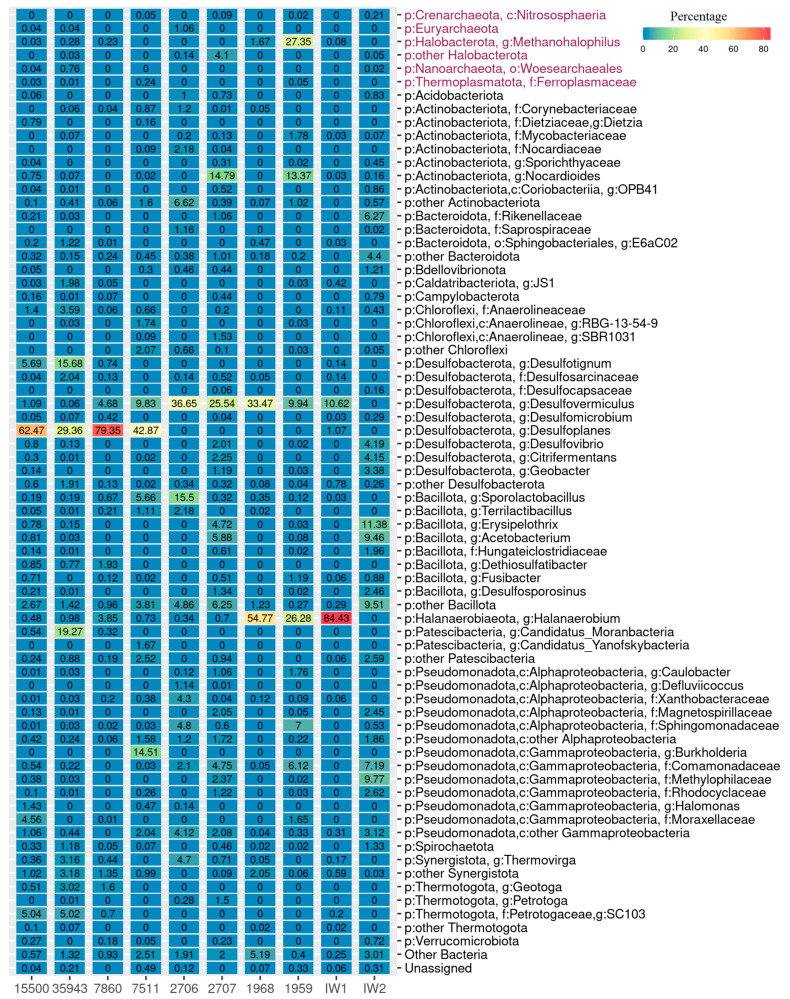
Heatmap of the composition of microbial communities in production and injection water samples from petroleum reservoirs based on 16S rRNA gene amplicon sequencing (SILVA Database). The values represent the relative abundance in the percentage of taxonomic groups in the sample. Archaea are marked in purple.

In all samples, predominant bacteria constituted at least 72% of the whole community (Figure 1). 

Members of the phyla *Desulfobacterota* (10.3–85.4%), *Bacillota* (1.6–22.5%), *Halanaerobiaeota* (0.3–54.8%), *Pseudomonadota* [classes *Alphaproteobacteria* (0.1–11.6%) and *Gammaproteobacteria* (0.01–17.3%)], and *Synergistota* (0.06–6.3%) were detected in high proportions in halophilic microbial communities from the eight studied PW samples. Members of the phyla *Actinobacteriota*, *Thermotogota*, *Halobacterota*, *Patescibacteria*, *Chloroflexi*, *Spirochaetota*, *Campylobacterota*, and *Bacteroidota* were detected in water from two to six production wells and represented minor microbial groups.

The composition of the community of the IW1 injection water was similar to that of the production water samples from oil fields with sandstone oil-bearing collectors. Fermentative bacteria of the genus *Halanaerobium* (84.4%), phylum *Halanaerobiaeota*, and sulfate-reducing bacteria of the genus *Desulfovermiculus*, phylum *Desulfobacterota* (12.8%), predominated in the IW1 sample, while in the IW2 sample the microbial community was more diverse and included members of the phyla *Bacillota* (35.7%), *Pseudomonadota* (class *Gammaproteobacteria*, 22.7%), *Desulfobacterota* (12.4%), and *Bacteroidota* (10.7%). 

At the genus level, the studied microbial communities included sulfate-reducing bacteria of the genera *Desulfoplanes*, *Desulfovermiculus*, and *Desulfotignum*, fermentative bacteria of the genera *Halanaerobium*, *Sporolactobacillus*, *Geotoga*, and *Thermovirga*, acetogenic bacteria of the genus *Acetobacterium*, uncultured bacteria of the family *Synergistaceae*, and methanogenic archaea of the genus *Methanohalophilus* (Figure 1). Bacteria of the genera *Nocardioides*, *Burkholderia*, and *Candidatus* Moranbacteria, and of the families *Methylophilaceae*, *Comamonadaceae*, and *Pseudomonadacea*, were also detected. 

Archaea were most abundant in sample PW1959, where they constituted 27.4% of the microbial community. In other samples, the share of archaea was below 1% (Figure 1). Archaeal sequences belonged mainly to methylotrophic halophilic methanogens of the genus *Methanohalophilus*, phylum *Halobacterota*.

**Figure 2:**
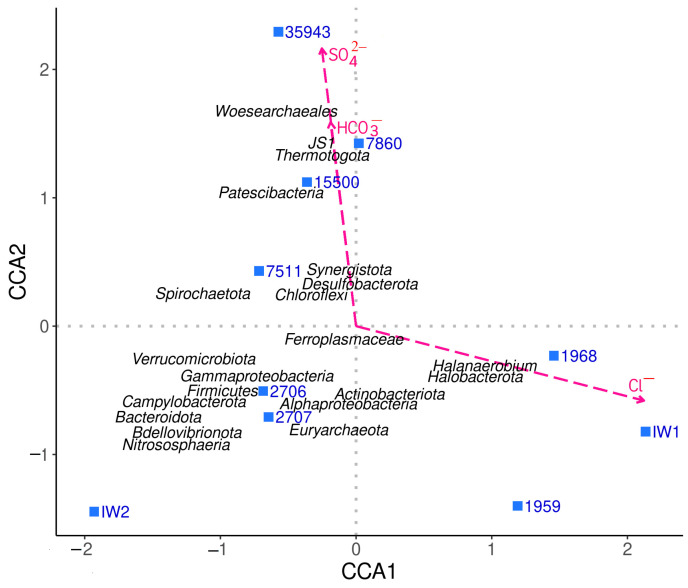
Canonical correlation analysis of the 16S rRNA gene-based diversity of prokaryotes and the geochemical parameters of production and injection water samples.

A comparison of 16S rRNA gene libraries using the principal component analysis (PCA) method revealed that the communities from the oil fields with carbonate collectors and high sulfate content (PW15500, PW35943, and PW7860) clustered together (Figure S2). The canonical correlation analysis (CCA) (Figure 2) confirmed the PCA distribution and the separate position of the oil field microbial communities from the carboniferous deposits. 

A correlation was established between high sulfate concentration and the occurrence of sulfate-reducing bacteria of the genera *Desulfoplanes* and *Desulfotignum*, as well as members of the phyla *Thermotogota* and *Synergistota*, in the reservoirs with carbonate collectors. The Devonian sandstone layers with higher salinity of formation water, high calcium content and low sulfate content were inhabited by halophilic methanogenic archaea of the genus *Methanohalophilus*, fermentative bacteria of the genus *Halanaerobium*, and sulfate-reducing bacteria of the genus *Desulfovermiculus*. 

### 3.3. Genomes Assembled from Microbial Communities’ Metagenomes

Metagenomes of microbial communities of formation water from production well PW15500 and of injection waters (IW1 and IW2) were sequenced using the Illumina HiSeq2500 platform for the analysis of the functional potential of microbial communities based on the metagenome-assembled genomes (MAGs). The analysis of the metagenome data resulted in the assembly of 16 (PW15500), 12 (IW1), and 47 (IW2) MAGs, among which 13, 6, and 32 MAGs, respectively, had a completeness over 50% and contamination below 15% (Tables S3–S5), according to the CheckM assessment of the presence of single-copy conserved genes. 

Genome bins representing these MAGs comprised 84.5%, 52.0%, and 40.5% of the whole metagenomes PW15500, IW1, and IW2, respectively. MAGs taxonomic classification according to the GTDB database [61] revealed the same major groups in all three metagenomes (PW15500, IW1, and IW2), which were detected by profiling the 16S rRNA gene fragments. 

**Figure 3:**
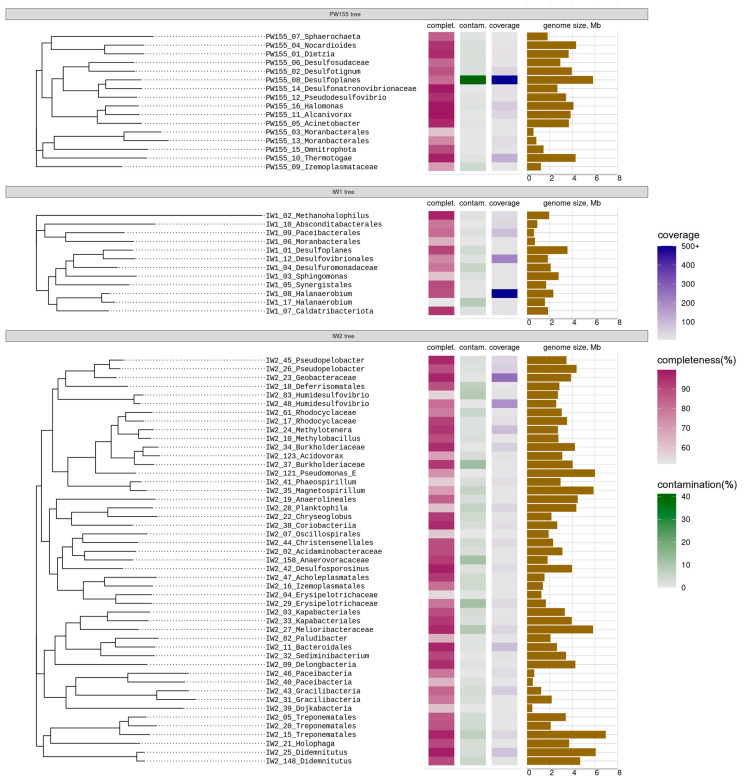
Genomes assembled from microbial communities’ metagenomes. MAGs assembled from formation water of production well 15,500 (designated as PW155) and from injection water samples IW1 and IW2.

In the metagenome of formation water PW15500, MAGs of bacteria of the phyla *Desulfobacterota* (PW155_08), *Pseudomonadota* (PW155_16), and *Thermotogota* (PW155_10) accounted for 77.4%, 1.2%, and 2.6% of the whole metagenome; they belonged to members of the genera *Desulfoplanes* and *Halomonas* and of the class *Thermotogae*, respectively (Table S3). The shares of other MAGs in the PW15500 metagenome were less than 0.8% each. MAG PW155_08 was close to the genome of the sulfate-reducing bacterium *Desulfoplanes formicivorans*. The PW155_16 genome belonged to a member of the genus *Halomonas*, class *Gammaproteobacteria*. MAG PW155_10 belonged to a member of the class *Thermotogae*, probably growing anaerobically via the fermentation of carbohydrates and proteins, as was confirmed by genomic data. The remaining MAGs in the PW15500 metagenome belonged to the minor components of the community, mainly to heterotrophic bacteria of the genera *Desulfotignum*, *Pseudodesulfovibrio*, *Sphaerochaeta*, *Alcanivorax*, *Dietzia*, and *Nocardioides*, and to uncultured bacteria of the phyla *Patescibacteria* and *Omnitrophota* (Figure 3).

In the IW1 metagenome, 49.2% of the sequences (Figure 3; Table S4) belonged to a member of the genus *Halanaerobium* (MAG IW1_08), phylum *Bacillota*. MAG IW1_12 (1.6%) belonged to a sulfate-reducing bacterium of the order *Desulfovibrionales*. MAG IW1_02 (0.23%) of a halophilic methylotrophic methanogenic archaeon of the genus *Methanohalophilus* was also assembled in the metagenome. 

Microorganisms of the IW2 sample exhibited the highest diversity (Figure 3; Table S5). The MAGs of members of the phylum *Desulfobacterota* constituted a significant part of the metagenome (*n* = 6, 15.3%); they belonged to sulfate-reducing bacteria, and all genes required for dissimilatory sulfate reduction were found in their genomes. Other genomes belonged to heterotrophic bacteria of the phyla *Bacteroidota* (*n* = 6, 4.95%) and *Actinobacteriota* (*n* = 3, 2.1%). Eight MAGs comprising 6.1% of the metagenome belonged to *Gammaproteobacteria* of the families *Burkholderiaceae* (*n* = 3), *Methylophilaceae* (*n* = 2), *Rhodocyclaceae* (*n* = 2), and *Pseudomonadaceae* (*n* = 1). The MAGs of the family *Methylophilaceae* belonged to methylotrophs capable of using methanol or methylamine as the sole source of carbon and energy. A number of microorganisms (genera *Erysipelothrix*, *Paludibacter*, *Candidatus* Planktophila, and *Candidatus* Didemnitutus) revealed by the metagenomic analysis of IW2 were aquatic bacteria often detected in contaminated surface river water [63,64,65].

### 3.4. The Genes Responsible for Hydrocarbon Degradation, Osmoprotection, and Sulfur Metabolism Revealed in MAGs

**Figure 4:**
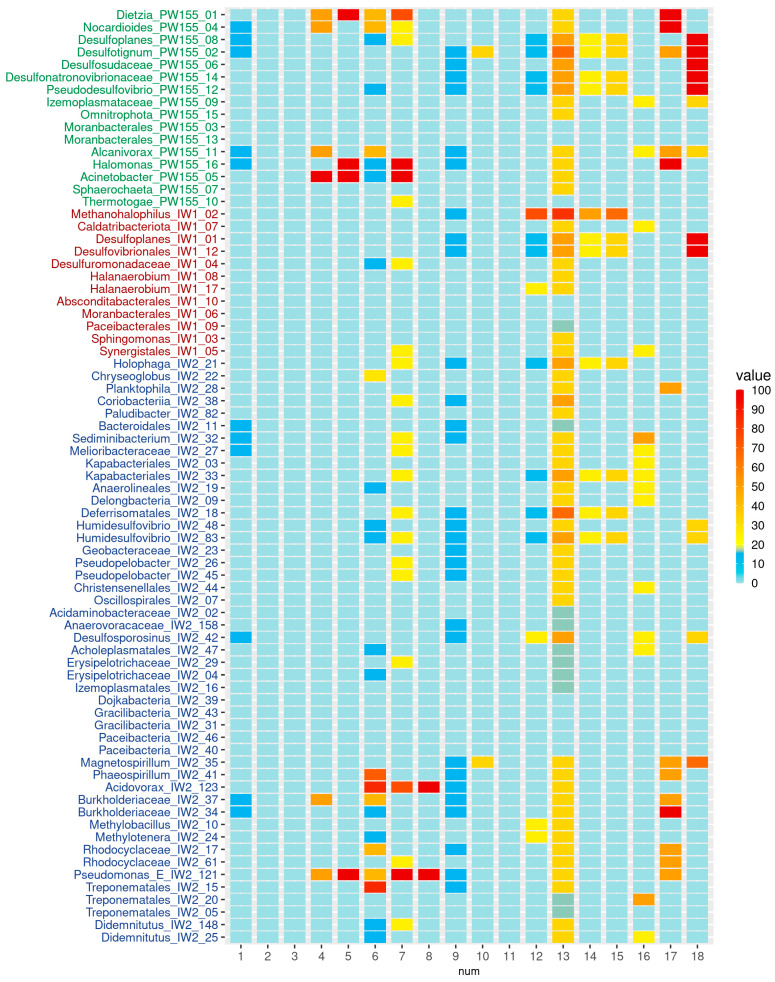
Heatmap showing KEGG module completeness in each MAG. Numbers below the bars: 1, ring cleavage via Baeyer–Villiger oxidation (K17754, K19960, K03379, K01053, K00002, K19961, K14519); 2, naphthalene degradation (M00534); 3, biphenyl degradation (M00543); 4, alkane-degradation (K00496, K20938); 5, benzoate degradation (M00551); 6, catechol meta-cleavage (M00569); 7, catechol ortho-cleavage (M00568); 8, benzene degradation, aerobic (M00548); 9, phenol degradation, anaerobic (K01612, K21759, K03186, K04105, K04107, K04108, K04109); 10, toluene degradation, anaerobic (M00418); 11, benzoyl-CoA degradation, anaerobic (M00541); 12, methanogenesis, CO_2_ (M00567); 13, methanogenesis, acetate (M00357); 14, methanogenesis, methylamines (M00563); 15, methanogenesis, methanol (M00356); 16, coenzyme M biosynthesis (M00358); 17, dissimilatory nitrate reduction (M00530); 18, dissimilatory sulfate reduction (M00596).

The genes of alkane 1-monooxygenase, determining the aerobic alkane degradation, were revealed in four genomes of the PW15500 microorganisms: *Dietzia maris* PW155_01, *Alcanivorax* PW155_11, *Nocardioides* PW155_04, and *Acinetobacter calcoaceticus* PW155_05 (Figure 4). 

Members of these genera are known to be able to degrade a broad spectrum of alkanes and aromatic hydrocarbons [66,67,68,69]. However, the genes encoding butane- and methane monooxygenases were not detected in the MAGs. Practically all assembled MAGs contained the genes responsible for osmoprotection. The genes of sulfur metabolism were mostly present in the MAGs of sulfate-reducing bacteria *Pseudodesulfovibrio* PW155_12, *Desulfonatronovibrionaceae* PW155_14, *Desulfotignum balticum* PW155_02, *Desulfosudaceae* PW155_06, and *Desulfoplanes* PW155_08. In the MAG *Halomonas* PW155_16, the operon of *ttrABC* genes responsible for tetrathionate reduction to thiosulfate was revealed, although the genes of the reverse process (*doxAD*) were not found. 

In MAGs from the IW1 sample, the genes responsible for hydrocarbon degradation were not revealed but, similar to most MAGs from PW15500, the genes protecting bacteria against high salinity were found. The genes for dissimilatory sulfate reduction were found in the MAGs *Desulfoplanes* IW1_01 and *Desulfovibrionales* IW1_12. In the MAGs *Pseudomonas* IW2_121, IW2_13, and IW2_160, the genes responsible for protection against high salinity and for the degradation of alkanes and aromatic hydrocarbon were found.

### 3.5. Diversity of Fermentative Enrichments and Production of Oil-Displacing Compounds

The population of cultured fermentative bacteria was predominant in the studied samples of production water. In this work, the ability of fermentative enrichments to produce metabolites promising for oil displacement was tested. Halophilic fermentative enrichments were obtained in media with 10% (*w*/*v*) NaCl and various organic substrates (starch, peptone, glycerol, and sucrose). By sequencing the V3–V4 region of the 16S rRNA genes, bacteria of the genera *Halanaerobium*, *Geotoga, Desulfoplanes*, *Desulfovermiculus*, and *Desulfuromonas*, and uncultured members of the families *Defluviitaleaceae* and *Synergistaceae* were revealed in fermentative enrichments (Figure S3). KEGG analysis predicted the potential activity of the enrichments in glycolysis and gluconeogenesis, as well as in pyruvate, propanoate, butanoate, galactose, fructose, methane, and sulfur metabolism (Figure S4). In cultivation experiments, enrichment obtained from PW15500 fermented a range of substrates with the formation of low fatty acids (acetic, propionic, and butyric), alcohol (ethanol), and gases (H_2_ and CO_2_) (Figure S5). The enrichment from the 7860 production well accumulated acetic, propionic, and butyric acids at concentrations of 556, 85 and 32 mg·L^−1^, respectively, when grown on peptone. Fermentative enrichments from the PW15500 production well accumulated acetic acid (220–428 mg·L^−1^) in media with starch, peptone, glucose, and glycerol. When these media were amended with thiosulfate as an electron acceptor, the PW15500 enrichment produced sulfide (Figure S5). In the medium with lactate and thiosulfate, the PW15500 enrichment produced acetic acid (29 mg·L^−1^) and sulfide (280 mg·L^−1^), indicating the presence of sulfidogenic bacteria incompletely oxidizing lactate. Moreover, in the medium with acetate and thiosulfate, this enrichment produced up to 405 mg sulfide per liter, supporting the suggestion that acetate-utilizing sulfidogens were also inhabiting this oil field. It is likely that in the presence of oxidized sulfur compounds and at a longer incubation time, sulfidogens would use acetate and other metabolic products of fermentative bacteria to form sulfide. It may be expected that the introduction of organic substrates into an oil reservoir will stimulate the growth of fermentative bacteria producing oil-displacing metabolites (lower acids and alcohols); the subsequent activity of sulfidogenic components of the community should, however, be taken into account.

### 3.6. Influence of Nitrate on Sulfidogenesis by Anaerobic Enrichments

Although the microorganisms inhabiting oil reservoirs are able to grow in the media for denitrifying/nitrate-reducing bacteria (DNB/NRB), due to the absence of nitrate in the oil fields, this process is usually not carried out, occurring only after nitrate injection into the reservoir [70,71]. Nitrate injection stimulates the growth of DNB/NRB and is one of the approaches used to suppress oil field sulfate-reducing bacteria (SRB) [1,72,73]. Since nitrate reduction is energetically more advantageous than sulfate reduction, DNB/NRB suppress the growth of SRB via competition for the same organic substrates. Nitrate reduction often results in the formation of nitrite, which inhibits sulfite reductase, suppressing sulfate-reducing and sulfite-reducing bacteria. Moreover, nitrite reacts chemically with sulfide, decreasing its concentration in formation water.

In the present work, the effect of nitrate on sulfide formation by the 15500_Pept fermentative enrichment in media with sulfate and thiosulfate was studied. This enrichment was obtained on the medium with peptone inoculated with water from well PW15500. The enrichment contained bacteria of the genera *Halanaerobium* (35.3%), *Geotoga* (20.2%), *Thermovirga* (16.2%), *Desulfoplanes* (7.5%), *Sulfurimonas* (2.1%), *Desulfuromonas* (0.9%), *Sphaerochaeta* (0.7%), and members of the family *Synergistaceae* (3.0%), of the orders *Peptostreptococcales*-*Tissierellales* (10.1%) and *Bacteroidales* (3.6%). If either sulfate or thiosulfate was present in the medium, the addition of nitrate (2 g·L^−1^) resulted in nitrite accumulation and decreased sulfide production (Figure S6).

### 3.7. Isolation and Characterization of Pure Cultures from Oil Fields

**Figure 5:**
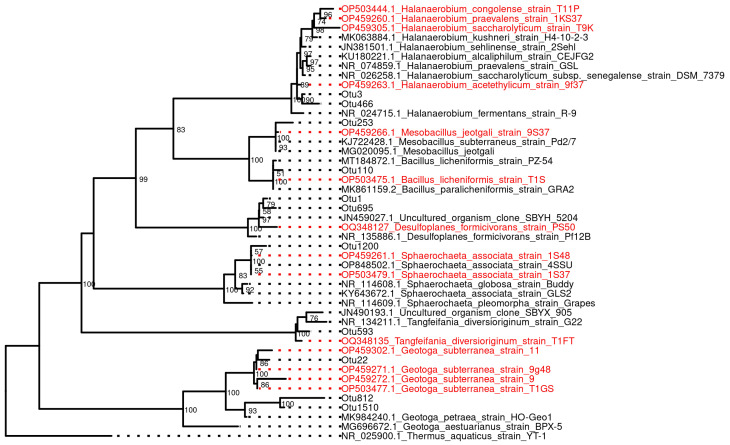
Phylogenetic tree of the V3–V4 hypervariable region of the 16S rRNA genes, including sequences of isolated pure cultures (marked in red) and the nearest representative sequences (marked in black) found in the course of the taxonomic analysis of microbial communities of PW and IW samples.

Enrichments obtained through the inoculation of formation water samples into the media for aerobic and anaerobic bacteria were used for the isolation of pure cultures. Through successive transfers of tenfold dilutions of the grown cultures into the relevant media, 20 strains were isolated (Table S6). The taxonomic affiliation of pure cultures was determined using 16S rRNA gene sequencing. The genes of 14 strains had 99.1–100% similarity with the 16S rRNA gene sequences of known species, and these strains were assigned to respective species (Figure 5).

Spore-forming isolates of the phylum *Bacillota* included *Bacillus licheniformis* (strains T1S and T21-1F), *Cytobacillus oceanisediminis* (T14KS and T21-11F), and *Mesobacillus jeotgali* (9S37, T9KN, T1KP). These strains were capable of growing in aerobic and anaerobic conditions, utilizing a range of substrates with oxygen or nitrate as electron acceptors. Anaerobic bacteria of the genus *Halanaerobium*, phylum *Bacillota*, predominant in of the many studied production water samples, were isolated and assigned to the known species *Halanaerobium acetethylicum* (9f37), *Halanaerobium congolense* (T11P), and *Halanaerobium praevalens* (1KS37). Strain T9K had a 98% 16S rRNA gene sequence similarity with *Halanaerobium saccharolyticum* subsp. *senegalense* and probably belongs to a new species of the *Halanaerobium* genus. Three strains (1S37, 1S48, and 4SSU) with spherical cells had 97.1–98.0% similarity between their 16S rRNA gene sequences and the sequence of *Sphaerochaeta associata,* phylum *Spirochaetota*, and represent a new species of the genus *Sphaerochaeta*. These strains fermented sucrose. Four strains, 9S, 11S, T1GS, and 9g48, whose 16S rRNA gene sequences had 99.7% similarity with that of *Geotoga subterranea* CC-1^T^, were isolated. The *G. subterranea* strains were represented by motile rods surrounded by sheaths. They fermented carbohydrates and proteins with the production of acetate, H_2_, and CO_2_, and reduced thiosulfate to sulfide. We succeeded in the isolation of the halophilic sulfate-reducing strain PS50 phylogenetically related to the bacterium *Desulfoplanes formicivorans* (98.6% similarity) and of the strain T1FT, probably representing a novel species of the genus *Tangfeifania* (98.5% similarity with *Tangfeifania diversioriginum*). The fragments of the 16S rRNA gene sequences similar to those of the isolated strains were present among the 16S rRNA OTUs from the samples of injected and formation water (Figure 5).

In our experiments, neither enrichments nor pure cultures of bacteria *C. oceanisediminis* T21-11F and *M. jeotgali* 9s37 grown in aerobic conditions showed a considerable decrease in the surface and interfacial tension; only the halophilic spore-forming bacterium *B. licheniformis* T21-1F exhibited a slight decrease in the interfacial tension when grown on molasses and peptone (Table S7).

## 4. Discussion

In this study, prokaryotic diversity in production and injection water samples from low-temperature petroleum reservoirs with high-saline formation water were investigated in order to predict the potential of these communities for the enhancement of oil recovery or the suppression of corrosion. Ten water samples collected from carbonate and sandstone oil-bearing collectors with different salinity levels and concentrations of sulfate and hydrogen sulfide in formation water were studied using cultural, 16S rRNA gene-based, and metagenomic (three samples) approaches.

### 4.1. Microbial Diversity in Oil Fields with Highly Saline Formation Water

Microbial communities of the Tatarstan oil reservoirs were adapted to the physicochemical conditions of their environment, with a high salinity of formation water, the presence of sulfate (and sometimes of sulfide, as well), and low temperature. Culture-based and 16S rRNA gene amplicon sequencing techniques revealed a predominance of fermentative and sulfate-reducing bacteria and the absence of aerobic organotrophic bacteria in most of the studied high-salinity formation water samples (Figure 1 and Figure S1). In the formation water (PW15500, PW35943, and PW7860) of the reservoirs with carbonate oil-bearing collectors, sulfate-reducing bacteria of the genera *Desulfoplanes* and *Desulfotignum* (phylum *Desulfobacterota*) predominated, and fermentative bacteria of the phyla *Thermotogota* (genera *Geotoga* and *Petrotoga*) and *Synergistota* (genus *Thermovirga*) were also present, as well as heterotrophic bacteria of the phyla *Pseudomonadota* (*Gammaproteobacteria* of the genera *Burkholderia* and *Halomonas*) and *Chloroflexi* (family *Anaerolineaceae*) and uncultured *Patescibacteria* (*Candidatus* Moranbacteria). The share of methanogenic archaea did not exceed 0.3% of the total microbial abundance (Figure 1; Table S8).

The composition of microbial communities in the reservoirs with sandstone collectors (PW1968 and PW1959) differed from that of carbonate collectors (Figure 1 and Figure 2). The formation water of Devonian oil deposits with sandstone collectors was characterized by a lower sulfate content and higher salinity. In production brines (PW1959, PW1968), the methanogenic archaea of the genus *Methanohalophilus* were revealed, with their relative abundance in the community being as high as 27.3%. In several samples, fermentative bacteria of the phylum *Halanaerobiaeota* (the genus *Halanaerobium*) and sulfate-reducing bacteria of the phylum *Desulfobacterota* (genus *Desulfovermiculus*, 9.9–36.6%) predominated; *Actinobacteriota* (genera *Nocardioides*, *Mycobacterium*, and *Rhodococcus*) and *Bacillota* (genus *Desulfosporosinus*) were also present. Spirochetes constituted minor groups in both types of oil collectors.

### 4.2. MAG-based Functional Predictions and Their Correspondence to the Features of Isolates

The results of 16S rRNA gene metabarcoding, which showed prokaryotic diversity in the oil fields studied, were supplemented by the analysis of metagenomes, which allowed the prediction of the metabolic potential of the components of the microbial community. In some cases, the results of bioinformatic analysis were confirmed by research on pure cultures. In total, 20 pure bacterial cultures of the genera *Desulfoplanes, Halanaerobium*, *Geotoga*, *Sphaerochaeta*, *Tangfeifania, Mesobacillus*, *Cytobacillus*, and *Bacillus* were isolated. Sulfidogenic bacteria included sulfate-reducing bacteria and fermentative bacteria of the genus *Halanaerobium*, which are capable of reducing oxidized sulfur compounds other than sulfate. In the studied reservoirs with high sulfate content in formation water, sulfate reduction was the dominant predicted process of oil biodegradation.

Metagenomes of sulfate-reducing bacteria of the genera *Desulfoplanes* and *Desulfotignum* were assembled from PW15500 and IW1 samples. MAG PW155_08, sequenced with the 2741-fold average coverage, was 5,852,592 bp long, and a BLAST comparison revealed its high similarity to the genome of the sulfate reducer *Desulfoplanes formicivorans*. The analysis of the MAG of *Desulfoplanes* sp. PW155_08 showed that this bacterium was probably capable of anaerobic sulfate reduction, with formate, lactate, and H_2_ as electron donors. The gene cluster encoding the dissimilatory nitrate reductase was also revealed.

At present, sulfate-reducing bacteria of the genus *Desulfoplanes* of the family *Desulfomicrobiaceae* comprise one species, *Desulfoplanes formicivorans*, isolated from the blackish sediment of a meromictic lake with high sulfide content in the water [74]. These mesophilic (growth range 13–50 °C), halophilic (NaCl range 0.5–8%, *w*/*v*), and neutrophilic bacteria utilize a narrow range of organic substrates (formate, fumarate, and lactate) and grow on H_2_ as an electron donor in the presence of sulfate. Organic substrates are incompletely oxidized to acetate. Thiosulfate and sulfite are also used as electron acceptors for growth. Pyruvate, malate, and fumarate are used for fermentative growth in the absence of acceptors. Members of the genus *Desulfoplanes* were also detected in production water from Brazilian petroleum reservoirs [21,75]. The halophilic sulfate-reducing strain PS50 isolated in our study was phylogenetically related to *Desulfoplanes formicivorans* (98.6% 16S rRNA gene sequence similarity) and demonstrated features of the species indicated above.

MAG PW155_02, which was sequenced with the 42.7-fold average coverage, was 3,957,006 bp long and BLAST comparison revealed its relation to the sulfate reducer *Desulfotignum balticum* of the family *Desulfobacteraceae.* Analysis of the MAG of *D. balticum* PW155_02 showed that this bacterium was incapable of using oxygen, but could grow with sulfite, sulfate, thiosulfate, nitrate, CO_2_, or fumarate as electron acceptors. For autotrophic growth, *D. balticum* PW155_02 may use the Wood–Lyundgahl pathway, all the required genes for which were detected. It is also capable of the anaerobic degradation of such aromatic compounds as toluene and benzoate. The gene cluster encoding the respiratory nitrate reductase was found in the genome.

Bacteria of the genera *Desulfoplanes* and *Desulfotignum*, incompletely oxidizing organic substrates to acetate and completely oxidizing to CO_2_, respectively, supplement each other in their metabolic characteristics. The genus *Desulfotignum* comprises three species of mesophilic bacteria growing in the media with 0.5–5/11% NaCl (*w*/*v*) [76,77,78]. In the presence of sulfate, *Desulfotignum balticum* and *Desulfotignum toluenicum* utilize a broad range of substrates, including simple organic compounds, fatty acids, and aromatic compounds, oxidizing them to CO_2_, and can also grow on H_2_ + CO_2_ and formate [76,78]. In addition to sulfate, thiosulfate and sulfite may be used as electron acceptors. In the absence of sulfate, they can ferment pyruvate. The genus also contains the ecologically valuable bacterium *Desulfotignum phosphitoxidans*, which grows lithoautotrophically with phosphite as an electron donor and sulfate as an electron acceptor [77]. In the absence of sulfate, phosphite oxidation was coupled to homoacetogenic acetate formation. Members of the genus *Desulfotignum* were isolated from marine sediments from an oil-reservoir model column, containing crude oil as the substrate, and were detected using molecular methods in low- and high-temperature petroleum reservoirs [79].

Although sulfate-reducing bacteria of the genus *Desulfovermiculus* were abundant in IW1, their MAGs were not assembled. Members of the genus *Desulfovermiculus* are mesophilic and grow in a broader salinity range (2–23% NaCl, *w*/*v*) than *Desulfoplanes* and *Desulfotignum*, which may explain their occurrence in higher salinity water. *Desulfovermiculus* spp. utilizes a wide range of organic substrates, which are completely oxidized in the process of sulfate reduction. The strain type of *Desulfovermiculus halophilus* was isolated from an oil reservoir in Vietnam [80]. These bacteria are also capable of autotrophic growth on H_2_ + CO_2_ in the presence of sulfate, and the fermentation of pyruvate and fumarate in the absence of sulfate.

Metagenome analysis and culture-based studies revealed that the SRB found in the reservoirs with saline water were characterized by their ability to carry out autotrophic sulfate reduction with molecular hydrogen and CO_2_.

Fermentative bacteria of the genera *Halanaerobium*, *Geotoga*, and *Thermovirga*, which were detected in the oil reservoirs in Tatarstan, have been reported as widely distributed groups in microbial communities of oil fields [75,81,82,83,84]. A member of the genus *Halanaerobium*, detected using 16S rRNA gene profiling (up to 54.7% sequences) in the samples from a sandstone oil reservoir, was also found through metagenome analysis. Two MAGs, IW1_08 and IW1_17, from the IW1 sample, were affiliated with *Halanaerobium* sp. and *Halanaerobium congolense*, respectively (Figure 3). MAG IW1_08, sequenced with the 5370.4-fold average coverage, was 2,315,003 bp long, and the BLAST comparison revealed its relation to the genome of the fermentative bacterium *Halanaerobium* sp. In the MAG IW1_08, 2293 numerous genes of hydrolases, degrading various sugars, were revealed.

Members of the genus *Halanaerobium* are known as anaerobic bacteria fermenting carbohydrates and are capable of reducing thiosulfate to sulfide [85]. They were detected using the molecular approach in water produced from the Marcellus and Barnett shale formations in the United States [82,86,87,88], in a Bakken shale oil field in Saskatchewan, Canada [81], and in the Vostochno-Anzirskoe oil field in Russia [84]. Bacteria *Halanaerobium salsuginis* (previously *Haloanaerobium salsugo*) [89] and *Halanaerobium kushneri* [90] were isolated from a sandstone oil reservoir in Oklahoma. *Halanaerobium congolense* was isolated from an offshore Congolese oil field in Africa [91]. The strain *Halanaerobium* sp. DL-01, isolated from a gas production field in the Barnett Shale (Arlington, TX, USA), degraded guar gum, used in fracture fluids, with the production of acetate and sulfide when thiosulfate was available [82]. Four *Halanaerobium* strains isolated in our study, 9f37, T11P, 1KS37, and T9K, were capable of growing on various sugars, on glycerol + peptone, and on fumarate (Table S3). In media with thiosulfate, fermentation products were changed in favor of acetate and sulfide, as was shown for enrichments (Figure S6).

Members of the phyla *Spirochaetota* were the minor components of microbial communities. MAG PW155_07 belonged to bacteria related to *Sphaerochaeta halotolerans* strain 4-11^T^ (ANI 99.15%, AAI 99.8%) isolated from an oil reservoir in Tatarstan [62]. MAG PW155_07, which was sequenced with 6.75-fold average coverage, was 1,826,087 bp long. In this MAG, the genes responsible for the reduction of sulfur compounds were not found. The *S. halotolerans* strains were anaerobic, fermentative, mesophilic, halotolerant bacteria; nitrate, sulfate, and thiosulfate were not reduced in mineral medium with maltose [62]. In this study, we isolated three strains, 1S37, 1S48, and 4SSU, probably belonging to a new species of the genus *Sphaerochaeta.* The genomes of strains 1S37 and 1S48 had 100% identity compared to each other, while the AAI with MAG PW155_07 was only 88.3%.

Isolated strains of the genus *Geotoga*, 9S, 11S, T1GS, and 9g48, reduced thiosulfate to sulfide in a medium with yeast extract and peptone. This observation shows that the oil reservoirs with highly saline formation water contained a phylogenetically diverse population of sulfidogenic bacteria. In the PW15500 metagenome, one MAG, PW155_10, was assigned to the phylum *Thermotogota*. Genome analysis did not reveal the complete set of genes responsible for the reduction of sulfur compounds.

MAG PW155_16, assembled in 104 contigs with the total length of 4,102,258 bp, belonged to a member of the genus *Halomonas*, class *Gammaproteobacteria*. Genome analysis revealed that this bacterium possessed the major characteristics of the genus and was a chemoorganotroph capable of using oxygen or nitrate as electron acceptors. The physiological properties of the strain *Halomonas titanicae* TAT1 [92], which has been previously isolated from oil reservoirs in Tatarstan, were in agreement with the characteristics predicted based on MAG PW155_16 analysis. The genome analysis of *H. titanicae* TAT1 revealed an absence of the *nirK* nitrite reductase gene, which explained the accumulation of ~100 mg of nitrite per liter during the anaerobic growth of the strain in the medium with acetate and nitrate. Nitrite is known to inhibit the growth of sulfidogens [1]. Batch cultures obtained by An and co-workers [73] from a Bakken shale oil field in Saskatchewan, Canada, showed sulfate-reducing and nitrate-reducing activities at salinities from 0 up to 2.5 M NaCl. Batch cultures accumulated nitrite in the media with high salinity, but not at low salinity. Bacteria of the genera *Halanaerobium* and *Desulfovermiculus* predominated at 2.5 M NaCl in the absence of nitrate, whereas high proportions of *Halomonas* and no SRB were found in the presence of nitrate [73]. These data demonstrate that similar microbial communities inhabit oil fields with high saline water in Tatarstan and in Canada and indicate the potential of *Halomonas* spp. for nitrate-mediated souring control at high salinity.

The only assembled archaeal MAG PW155_02 belonged to a methylotrophic methanogen, *Methanohalophilus euhalobius.* This genome comprises the genes for glycine betaine synthesis and for glycine betaine transport from the environment into the cell; they are involved in osmoadaptation, and their presence indicated a good adaptation of the archaeon to the conditions of its habitat [93]. The strain type of this species was originally isolated from a Tatarstan oil reservoir and described by Obraztsova et al. [94] as *Methanococcoides euhalobius*, later reclassified as *Methanohalophilus euhalobius* by Davidova et al. [6]. The strain formed biofilms, which depended on the presence of calcium in a medium. Members of the genus *Methanohalophilus* were also isolated from oil reservoirs [95], gas-bearing deep aquifers [96], saline lakes [97], and a deep hypersaline anoxic basin [98]. This genus comprises halophilic, methylotrophic methanogens; they utilize methylamines that are produced in the course of the decomposition of osmoregulatory substances [99]. For *Methanohalophilus profundi*, piezophilic growth at 35 Mpa (14 °C) was shown [98].

These results indicate the predominance of sulfidogenic prokaryotes in reservoirs with highly saline water; they are both sulfate reducers and fermentative bacteria capable of reducing oxidized sulfur compounds other than sulfate. In the studied reservoirs with high sulfate content in formation water, sulfate reduction was the dominant process of oil biodegradation, while in sandstone oil collectors with low-sulfate formation water halophilic methylotrophic archaea capable of methanogenesis were present.

### 4.3. Biotechnological Potential of Prokaryotes in High-Salinity Petroleum Reservoirs

Metagenomic and culture-based studies revealed no populations of aerobic organotrophic bacteria and a predominance of fermentative and sulfate-reducing bacteria in formation water samples. The low abundance of aerobic oil-oxidizing bacteria and high sulfide content in the formation water of deposit 302 of the Romashkinskoe oil field indicates that the application of microbial technologies for enhanced oil recovery based on oil biodegradation under these conditions is not reasonable. Injected oxygen will react with sulfide dissolved in the formation water, which may lead to the deposition of elemental sulfur and the plugging of the near-bottom zone of injection wells.

The activation of the fermentative microbiota via the injection of organic substrates from the surface looks a more promising approach in the case of reservoirs with high salinity and high levels of sulfate and sulfide.

The world’s largest trial of MEOR technology based on the introduction of *Clostridium tyrobutyricum* and molasses into the oil field was previously carried out on the H_2_S-rich bed 302 with the carbonate oil-bearing rock of the Romashkinskoe oil field [41]. In the reservoir, bacteria produced a broad spectrum of oil-displacing metabolites, which resulted in the additional recovery of over 4.5 t oil per 1 t of added molasses. The study of the 302 bed was the first experimental demonstration of an oil reservoir being an integral ecosystem, in which the biotic community interacted with the abiotic environment so that the energy flow created a specific trophic structure. The energy flows were based on the biotransformation of oil or exogenous substrates in a defined trophic chain and may be intentionally regulated.

The trial of MEOR technology based on oil degradation at the small lot of the Cheremukhovskoe oil field with highly saline water and low sulfate content was successful [42]. This reservoir contained no sulfide, which hinders the growth of aerobic bacteria. The injection of oil-oxidizing bacteria adapted to oil field conditions, as well as the addition of oxygen (as an H_2_O_2_ solution) and of the (NH_4_)_2_HPO_4_ solution as a source of biogenic elements, resulted in the recovery of 1141 t of additional oil.

The results of culture-based and metagenomic research on the microorganisms from oil reservoirs with high-salinity formation water were complementary and showed the necessity of a search for promising halophilic fermentative bacteria, producers of oil-displacing metabolites, and for the selection of cheap substrates for MEOR technologies suitable for specific reservoirs, as well as the need for the combination of these technologies with the suppression of sulfidogens.

## 5. Conclusions

The number of metagenomes related to petroleum reservoirs is still limited compared with the 16S rRNA profiling data. Metagenome analysis and cultural studies of microorganisms inhabiting mesothermic petroleum reservoirs with highly saline water were conducted to identify microorganisms with the potential for MEOR or the suppression of corrosion. Data obtained using 16S rRNA gene profiling and metagenomes sequencing were congruent at the phylum and genus level in the studied samples. The β-diversity of prokaryotes was driven by the sulfate content and salinity of formation water. The data highlighted the importance of sulfidogenic bacteria of the genera *Desulfoplanes*, *Desulfovermiculus*, *Desulfotignum*, and *Halanaerobium* in the studied petroleum reservoirs. Methylotrophic methanogens of the genus *Methanohalophilus* inhabited reservoirs with high-saline water and low sulfate content. A total of 75 metagenome-assembled genomes (MAGs) of prokaryotes were recovered, among which were taxa potentially representing novel species or genus levels. These MAGs displayed an abundance of genes conferring resistance to high salinity, the degradation of aromatic compounds, sulfate reduction, and hydrogenotrophic, acetotrophic and methylotrophic methanogenesis.

The low abundance of aerobic bacteria and high hydrogen sulfide content in reservoirs with carbonate collectors (such as bed 302 of the Romashkinskoe oil field) indicated that MEOR technologies based on oil oxidation cannot be applied with these collectors. The activation of fermentative bacteria via the injection of sugar-containing and/or proteinaceous substrates can be a more preferrable approach for these oil reservoirs. Cultures of halophilic fermentative bacteria inhabiting these oil reservoirs are capable of producing oil-displacing metabolites with lower fatty acids, alcohols, and gases (CO_2_ and H_2_) from sugar-containing and proteinaceous substrates, which testifies to their biotechnological potential for MEOR. However, when choosing a substrate for the activation of fermenting bacteria, the possibility of the growth of sulfate-reducing bacteria should be taken into account. The well-known technology of nitrate injection in high concentrations (about 2 g·L^−1^) can be used to suppress the growth of sulfidogens in the reservoirs. The results obtained from the metagenomic analysis can be verified in the course of future studies of a large collection of bacteria isolated from oil reservoirs.

## Figures and Tables

**Table 1:** Physicochemical characteristics of injection and production water samples from petroleum reservoirs.

Oil Field, Well	Total Salinity, g L^−1^	pH	Content, g L^−1^						Acetate, mg L^−1^
			Na^+^ + K^+^	Ca^2+^	Mg^2+^	Cl^−^	HCO_3_^−^	SO_4_^2−^	
Romashkinskoe									
PW15500 *	75.8533	7.55	24.0550	3.0060	1.7020	42.5440	0.5120	4.0343	3.2
PW35943 *	62.5517	7.52	22.0020	1.4030	0.608	31.9080	0.2680	6.3627	0
Arkhangelskoe									
PW7860 *	116.3550	6.52	36.7690	6.4130	1.8240	70.1970	0.3840	0.7680	1.8
IW2	192.6470	6.2	59.5190	10.0200	4.3780	117.7040	0.2200	0.8060	11.0
Novo-Elhovskoe									
PW7511	107.2180	6.11	30.2490	7.3590	2.9770	65.9430	0.3900	0.3000	4.4
PW2706	97.6970	6.47	26.4370	8.7900	1.9850	60.2700	0.1950	0.0200	0
PW2707	138.6900	6.55	38.4240	8.8180	4.8640	86.5050	0.0730	0.0060	7.0
Sabanchinskoe									
IW1	165.9610	6.55	55.0580	7.7580	2.8230	99.8770	0.2250	0.2250	5.5
PW1959	156.8480	6.73	48.3090	9.2180	2.9180	95.7230	0.2320	0.4480	5.9
PW1968	157.8584	6.52	46.1510	8.4170	5.1070	97.4960	0.2320	0.4554	6.1
Standard error, mg L^−1^		±0.1	±0.02	±0.09	±0.09	±0.004	±0.001	±0.02	±0.1

* Water samples from petroleum reservoirs with carbonate collectors.

## Data Availability

The raw data generated from 16S rRNA gene profiling and metagenome sequencing and MAG assemblies are accessible via the BioProject PRJNA927132. Nucleotide sequences of the 16S rRNA gene of pure cultures were deposited into GenBank under accession nos. OQ348127, OQ348135, OP459260, OP459261, OP459263, OP459266, OP459271, OP459272, OP459302, OP459305, OP503444, OP503475, OP503477, OP503479, OP588951, OP848502, OP849624, OP849652, and OP849684.

## Supplementary Materials

The following supporting information can be downloaded at: https://www.mdpi.com/article/10.3390/biology12101300/s1, Figure S1. Numbers of aerobic and anaerobic microorganisms in injection and production water samples from oil fields located in Tatarstan (Russia). Designations: AOB, aerobic organotrophic bacteria; FERM, fermentative bacteria; DNB, denitrifying bacteria; SRB, sulfate-reducing bacteria; MET, methanogenic archaea. Samples of injection water are designated as IW1 and IW2, and the rest are water samples from production wells; Figure S2. Principle components analysis (PCA) of the composition of microbial communities of injection and production water from oil fields of Tatarstan, based on the relative abundance of operational taxonomic units (≥97% similarity of 16S pPHK gene sequences) of prokaryotes; Figure S3. Heatmap of the relative abundance of bacteria at the genus level based on 16S rRNA amplicon sequencing (SILVA Database) in fermentative enrichments obtained from the production and injection water samples. Representation of the genus was calculated as sequence proportions divided by total sequence count in each library. Columns are clustered using correlation distance and average linkage. Enrichments were obtained in media with starch, peptone (Pept), sucrose (Sucr), and glycerol (Glyc); Figure S4. Heatmap showing metabolic pathways (KEGG Database) in fermentative enrichments obtained from the production and injection water samples; Figure S5. Concentrations of low fatty acids and sulfide in a medium (A) and CO_2_ and H_2_ in a gas phase (%, *v*/*v*; B) produced through PW15500 fermentative enrichment in media with various organic substrates amended with sodium thiosulfate or without it after 14 days of incubation at 37 °C; Figure S6. Production of sulfide (A) and nitrite (B) by PW15500_Pept fermentative enrichment in media with peptone and thiosulfate or sulfate and different initial nitrate concentrations. Enrichments were incubated at 22 °C for 28 days. Table S1. Characteristics of the oil reservoirs; Table S2. Number of sequences and diversity indices in the libraries of the 16S rRNA gene fragments of microorganisms in injection and production water samples from oil fields located in Tatarstan; Table S3. General characteristics of MAGs in production water sample 15500 (PW15500); Table S4. General characteristics of MAGs in injection water sample 1 (IW1); Table S5. General characteristics of MAGs in injection water sample 2 (IW2); Table S6. Taxonomic affiliation of pure cultures isolated from production and injection water samples; Table S7. Surface tension (ST) and interfacial tension (IT) of culture liquid with bacteria grown on various substrates; Table S8. Taxonomic analysis of PW and IW samples in aerobic conditions.

## References

[1] Gieg L.M., Jack T.R., Foght J.M. (2011). Biological souring and mitigation in oil reservoirs. Appl. Microbiol. Biotechnol..

[2] Head I.M., Gray N.D., Larter S.R. (2014). Life in the slow lane; biogeochemistry of biodegraded petroleum containing reservoirs and implications for energy recovery and carbon management. Front. Microbiol..

[3] Wilhelms A., Larter S.R., Head I., Farrlmond P., di-Primio R., Zwach C. (2001). Biodegradation of oil in uplifted basins prevented by deep-burial sterilization. Lett. Nat..

[4] Magot M., Ollivier B., Patel B.K.C. (2000). Microbiology of petroleum reservoirs. Antonie Van Leeuwenhoek.

[5] Liang R., Grizzle R.S., Duncan K.E., McInerney M.J., Suflita J.M. (2014). Roles of thermophilic thiosulfate-reducing bacteria and methanogenic archaea in the biocorrosion of oil pipelines. Front. Microbiol..

[6] Davidova I.A., Harmsen H.J., Stams A.J., Belyaev S.S., Zehnder A.J.B. (1997). Taxonomic description of *Methanococcoides euhalobius* and its transfer to the *Methanohalophilus* genus. Antonie Van Leeuwenhoek.

[7] Zhao J.-Y., Hu B., Dolfing J., Li Y., Tang Y.-Q., Jiang Y., Chi C.-Q., Xing J., Nie Y., Wu X.-L. (2021). Thermodynamically favorable reactions shape the archaeal community affecting bacterial community assembly in oil reservoirs. Sci. Total Environ..

[8] An D., Caffrey S.M., Soh J., Agrawal A., Brown D., Budwill K., Dong X., Dunfield P.F., Foght J., Gieg L.M. (2013). Metagenomics of hydrocarbon resource environments indicates aerobic taxa and genes to be unexpectedly common. Environ. Sci. Technol..

[9] Nie Y., Zhao J.-Y., Tang Y.-Q., Guo P., Yang Y., Wu X.-L., Zhao F. (2016). Species divergence vs. functional convergence characterizes crude oil microbial community assembly. Front. Microbiol..

[10] Liu Y.F., Galzerani D.D., Mbadinga S.M., Zaramela L.S., Gu J.D., Mu B.Z., Zengler K. (2018). Metabolic capability and in situ activity of microorganisms in an oil reservoir. Microbiome.

[11] Wang X., Li X., Yu L., Huang L., Xiu J., Lin W., Zhang Y. (2019). Characterizing the microbiome in petroleum reservoir flooded by different water sources. Sci. Total Environ..

[12] Song Z., Chen S., Zhao F., Zhu W. (2019). Whole metagenome of injected and produced fluids reveal the heterogenetic characteristics of the microbial community in a water-flooded oil reservoir. J. Pet. Sci. Eng..

[13] Jiao Y., An L., Wang W., Ma J., Wu C., Wu X. (2023). Microbial communities and their roles in the Cenozoic sulfurous oil reservoirs in the Southwestern Qaidam Basin, Western China. Sci. Rep..

[14] Hu P., Tom L., Singh A., Thomas B.C., Baker B.J., Piceno Y.M., Andersen G.L., Banfield J.F. (2016). Genome-resolved metagenomic analysis reveals roles for candidate phyla and other microbial community members in biogeochemical transformations in oil reservoirs. mBio.

[15] Vigneron A., Alsop E.B., Lomans B.P., Kyrpides N.C., Head I.M., Tsesmetzis N. (2017). Succession in the petroleum reservoir microbiome through an oil field production lifecycle. ISME J..

[16] Sierra-Garcia I.N., Dellagnezze B.M., Santos V.P., Chaves B M.R., Capilla R., Santos Neto E.V., Gray N., Oliveira V.M. (2017). Microbial diversity in degraded and non-degraded petroleum samples and comparison across oil reservoirs at local and global scales. Extremophiles.

[17] Hidalgo K.J., Sierra-Garcia I.N., Zafra G., de Oliveira V.M. (2021). Genome-resolved meta-analysis of the microbiome in oil reservoirs worldwide. Microorganisms.

[18] Guan J., Zhang B.L., Mbadinga S.M., Liu J.F., Gu J.D., Mu B.Z. (2014). Functional genes (*dsr*) approach reveals similar sulphidogenic prokaryotes diversity but different structure in saline waters from corroding high temperature petroleum reservoirs. Appl. Microbiol. Biotechnol..

[19] Gao P., Tian H., Wang Y., Li Y., Li Y., Xie J., Zeng B., Zhou J., Li G., Ma T. (2016). Spatial isolation and environmental factors drive distinct bacterial and archaeal communities in different types of petroleum reservoirs in China. Sci. Rep..

[20] Xiao M., Sun S.S., Zhang Z.Z., Wang J.M., Qiu L.W., Sun H.Y., Song Z.Z., Zhang B.Y., Gao D.L., Zhang G.Q. (2016). Analysis of bacterial diversity in two oil blocks from two low-permeability reservoirs with high salinities. Sci. Rep..

[21] Pereira G.F., Pilz-Junior H.L., Corção G. (2021). The impact of bacterial diversity on resistance to biocides in oilfields. Sci. Rep..

[22] Scheffer G., Hubert C.R.J., Enning D.R., Lahme S., Mand J., de Rezende J.R. (2021). Metagenomic investigation of a low diversity, high salinity offshore oil reservoir. Microorganisms.

[23] Piceno Y.M., Reid F.C., Tom L.M., Conrad M.E., Bill M., Hubbard C.G., Fouke B.W., Graff C.J., Han J., Stringfellow W.T. (2014). Temperature and injection water source influence microbial community structure in four Alaskan North Slope hydrocarbon reservoirs. Front. Microbiol..

[24] Li X.X., Mbadinga S.M., Liu J.F., Zhou L., Yang S.Z., Gu J.D., Mu B.Z. (2017). Microbiota and their affiliation with physiochemical characteristics of different subsurface petroleum reservoirs. Int. Biodeterior. Biodegrad..

[25] Sokolova D.S., Semenova E.M., Grouzdev D.S., Bidzhieva S.K., Babich T.L., Loiko N.G., Ershov A.P., Kadnikov V.V., Beletsky A.V., Mardanov A.V. (2021). Sulfidogenic microbial communities of the Uzen high-temperature oil field in Kazakhstan. Microorganisms.

[26] Xu Y., Wang J., Liu Q., Zhang Q., Wu J., Zhou M., Nie Y., Wu X.-L. (2023). pH and nitrate drive bacterial diversity in oil reservoirs at a localized geographic scale. Microorganisms.

[27] Orphan V.J., Taylor L.T., Hafenbradl D., Delong E.F. (2000). Culture-dependent and culture-independent characterization of microbial assemblages associated with high-temperature petroleum reservoirs. Appl. Environ. Microbiol..

[28] Grabowski A., Nercessian O., Fayolle F., Blanchet D., Jeanthon C. (2005). Microbial diversity in production waters of a low-temperature biodegraded oil reservoir. FEMS Microbiol. Ecol..

[29] Gieg L.M., Davidova I.A., Duncan K.E., Suflita J.M. (2010). Methanogenesis, sulfate reduction and crude oil biodegradation in hot Alaskan oilfields. Environ. Microbiol..

[30] Nazina T.N., Shestakova N.M., Semenova E.M., Korshunova A.V., Kostrukova N.K., Tourova T.P., Min L., Feng Q., Poltaraus A.B. (2017). Diversity of metabolically active Bacteria in water-flooded high-temperature heavy oil reservoir. Front. Microbiol..

[31] Belyaev S.S., Laurinavichus K.S., Obraztsova A.Y., Gorlatov S.N., Ivanov M.V. (1982). Microbiological processes in the near-bottom zone of injection wells of oil fields. Microbiology.

[32] Ivanov M.V., Belyaev S.S., Donaldson E.C. (1991). Biotechnology of Enhancement of Oil Recovery Based on the Geochemical Activity of Microorganisms (Field Experiments), Microbial Enhancement of Oil Recovery—Recent Advances.

[33] Lenchi N., İnceoğlu Ö., Kebbouche-Gana S., Gana M.L., Llirós M., Servais P., García-Armisen T. (2013). Diversity of microbial communities in production and injection waters of Algerian oilfields revealed by 16S rRNA gene amplicon 454 pyrosequencing. PLoS ONE.

[34] Belyaev S.S., Borzenkov I.A. (1993). Microbial transformation of low-molecular-weight carbon compounds in the deep subsurface. Biogeochemistry of Global Change.

[35] Nazina T.N., Shestakova N.M., Ivoilov V.S., Kostrukova N.K., Belyaev S.S., Ivanov M.V. (2017). Radiotracer assay of microbial processes in petroleum reservoirs. Adv. Biotech. Microbiol..

[36] Belyaev S.S., Borzenkov I.A., Nazina T.N., Rozanova E.P., Glumov I.F., Ibatullin R.R., Ivanov M.V. (2004). Use of microorganisms in the biotechnology for the enhancement of oil recovery. Microbiology.

[37] Youssef N., Elshahed M.S., McInerney M.J. (2009). Microbial processes in oil fields: Culprits, problems and opportunities. Adv. Appl. Microbiol..

[38] Gao G., Ji K., Zhang Y., Liu X., Dai X., Zhi B., Cao Y., Liu D., Wu M., Li G. (2020). Microbial enhanced oil recovery through deep profile control using a conditional bacterial cellulose-producing strain derived from *Enterobacter* sp. FY-07. Microb. Cell Factories.

[39] Wu B., Xiu J., Yu L., Huang L., Yi L., Ma Y. (2022). Research advances of microbial enhanced oil recovery. Heliyon.

[40] Ivanov M.V., Belyaev S.S., Borzenkov I.A., Glumov I.F., Ibatullin R.R. (1993). Additional oil production during field trials in Russia. Dev. Pet. Sci..

[41] Nazina T.N., Ivanova A.E., Ivoilov V.S., Miller Y.M., Kandaurova G.F., Ibatullin R.R., Belyaev S.S., Ivanov M.V. (1999). Results of the trial of the microbiological method for the enhancement of oil recovery at the carbonate collector of the Romashkinskoe oil field: Biogeochemical and production characteristics. Microbiology.

[42] Nazina T., Sokolova D., Grouzdev D., Semenova E., Babich T., Bidzhieva S., Serdukov D., Volkov D., Bugaev K., Ershov A. (2020). The potential application of microorganisms for sustainable petroleum recovery from heavy oil reservoirs. Sustainability.

[43] Nazina T.N., Shestakova N.M., Pavlova N.K., Tatarkin Y.V., Ivoilov V.S., Khisametdinov M.R., Sokolova D.S., Babich T.L., Tourova T.P., Poltaraus A.B. (2013). Functional and phylogenetic microbial diversity in formation waters of a low-temperature carbonate petroleum reservoir. Int. Biodeterior. Biodegradation.

[44] Kadnikov V.V., Frank Y.A., Mardanov A.V., Beletskii A.V., Ivasenko D.A., Pimenov N.V., Karnachuk O.V., Ravin N.V. (2017). Uncultured bacteria and methanogenic archaea predominate in the microbial community of Western Siberian deep subsurface aquifer. Microbiology.

[45] Semenova E.M., Ershov A.P., Sokolova D.S., Tourova T.P., Nazina T.N. (2020). Diversity and biotechnological potential of nitrate-reducing bacteria from heavy-oil reservoirs (Russia). Microbiology.

[46] Trüper H.G., Schlegel H.G. (1964). Sulfur metabolism in *Thiorhodaceae*. I. Quantitative measurements on growing cells of *Chromatium okenii*. Antonie Van Leeuwenhoek.

[47] Frey B., Rime T., Phillips M., Stierli B., Hajdas I., Widmer F., Hartmann M. (2016). Microbial diversity in European alpine permafrost and active layers. FEMS Microbol. Ecol..

[48] Magoč T., Salzberg S.L. (2011). FLASH: Fast length adjustment of short reads to improve genome assemblies. Bioinformatics.

[49] Edgar R.C. (2010). Search and clustering orders of magnitude faster than BLAST. Bioinformatics.

[50] Rognes T., Flouri T., Nichols B., Quince C., Mahé F. (2016). VSEARCH: A versatile open source tool for metagenomics. PeerJ.

[51] Martin M. (2011). Cutadapt removes adapter sequences from high-throughput sequencing reads. EMBnet J..

[52] Kang D.D., Froula J., Egan R., Wang Z. (2015). MetaBAT, an efficient tool for accurately reconstructing single genomes from complex microbial communities. PeerJ.

[53] Wu Y.-W., Simmons B.A., Singer S.W. (2016). MaxBin 2.0: An automated binning algorithm to recover genomes from multiple metagenomic datasets. Bioinformatics.

[54] Alneberg J., Bjarnason B.S., de Bruijn I., Schirmer M., Quick J., Ijaz U.Z., Lahti L., Loman N.J., Andersson A.F., Quince C. (2014). Binning metagenomics contigs by coverage and composition. Nat. Methods.

[55] Parks D.H., Imelfort M., Skennerton C.T., Hugenholtz P., Tyson G.W. (2015). CheckM: Assessing the quality of microbial genomes recovered from isolates, single cells, and metagenomes. Genome Res..

[56] Chaumeil P.-A., Mussig A.J., Hugenholtz P., Parks D.H. (2020). GTDB-Tk: A toolkit to classify genomes with the Genome Taxonomy Database. Bioinformatics.

[57] Parks D.H., Chuvochina M., Waite D.W., Rinke C., Skarshewski A., Chaumeil P.A., Hugenholtz P. (2018). A standardized bacterial taxonomy based on genome phylogeny substantially revises the tree of life. Nat. Biotechnol..

[58] Palù M., Basile A., Zampieri G., Treu L., Rossi A., Morlino M.S., Campanaro S. (2022). KEMET–A python tool for KEGG Module evaluation and microbial genome annotation expansion. Comput. Struct. Biotechnol. J..

[59] Koch A.L., Gerhardt P., Murray R.G.E., Wood W.A., Krieg N.R. (1994). Most probable numbers. Methods for General and Molecular Bacteriology.

[60] Pfennig N., Lippert K.D. (1966). Über das vitamin B12—Bedürfnis phototropher Schweferelbakterien. Arch. Microbiol..

[61] Wolin E.A., Wolin M.J., Wolfe R.S. (1963). Formation of methane by bacterial extracts. J. Biol. Chem..

[62] Bidzhieva S.K., Sokolova D.S., Grouzdev D.S., Kostrikina N.A., Poltaraus A.B., Tourova T.P., Shcherbakova V.A., Troshina O.Y., Nazina T.N. (2020). *Sphaerochaeta halotolerans* sp. nov., a novel spherical halotolerant spirochete from a Russian heavy oil reservoir, emended description of the genus *Sphaerochaeta*, reclassification of *Sphaerochaeta coccoides* to a new genus *Parasphaerochaeta* gen. nov. as *Parasphaerochaeta coccoides* comb. nov. and proposal of *Sphaerochaetaceae* fam. nov. Int. J. Syst. Evol. Microbiol..

[63] Jezbera J., Sharma A.K., Brandt U., Doolittle W.F., Hahn M.W. (2009). ‘*Candidatus* Planktophila limnetica’, an actinobacterium representing one of the most numerically important taxa in freshwater bacterioplankton. Int. J. Syst. Evol. Microbiol..

[64] Lopera J., Miller I.J., McPhail K.L., Kwan J.C. (2017). Increased biosynthetic gene dosage in a genome-reduced defensive bacterial symbiont. mSystems.

[65] Eisenberg T., Muhldorfer K., Erhard M., Fawzy A., Kehm S., Ewers C., Semmler T., Blom J., Lipski A., Rau J. (2022). *Erysipelothrix anatis* sp. nov., *Erysipelothrix aquatica* sp. nov. and *Erysipelothrix urinaevulpis* sp. nov., three novel species of the genus, and emended description of *Erysipelothrix*. Int. J. Syst. Evol. Microbiol..

[66] Gregson B.H., Metodieva G., Metodiev M.V., McKew B.A. (2019). Differential protein expression during growth on linear versus branched alkanes in the obligate marine hydrocarbon-degrading bacterium *Alcanivorax borkumensis* SK2^T^. Environ. Microbiol..

[67] Ho M.T., Li M.S.M., McDowell T., MacDonald J., Yuan Z.C. (2020). Characterization and genomic analysis of a diesel-degrading bacterium, *Acinetobacter calcoaceticus* CA16, isolated from Canadian soil. BMC Biotechnol..

[68] Venil C.K., Malathi M., Devi P.R. (2021). Characterization of *Dietzia maris* AURCCBT01 from oil-contaminated soil for biodegradation of crude oil. 3 Biotech..

[69] Mitzscherling J., MacLean J., Lipus D., Bartholomäus A., Mangelsdorf K., Lipski A., Roddatis V., Liebner S., Wagner D. (2022). *Nocardioides alcanivorans* sp. nov., a novel hexadecane-degrading species isolated from plastic waste. Int. J. Syst. Evol. Bacteriol..

[70] Bødtker G., Lysnes K., Torsvik T., Bjørnestad E.Ø., Sunde E. (2009). Microbial analysis of backflowed injection water from a nitrate-treated North Sea oil reservoir. J. Ind. Microbiol. Biotechnol..

[71] Gittel A., Sorensen K.B., Skovhus T.L., Ingvorsen K., Schramm A. (2009). Prokaryotic community structure and activity of sulfate reducers in production water from high-temperature oil reservoirs with and without nitrate treatment. Appl. Environ. Microbiol..

[72] Fida T.T., Gassara F., Voordouw G. (2017). Biodegradation of isopropanol and acetone under denitrifying conditions by *Thauera* sp. TK001 for nitrate-mediated microbially enhanced oil recovery. J. Hazard. Mater..

[73] An B.A., Shen Y., Voordouw G. (2017). Control of sulfide production in high salinity Bakken Shale oil reservoirs by halophilic bacteria reducing nitrate to nitrite. Front. Microbiol..

[74] Watanabe M., Kojima H., Fukui M. (2015). *Desulfoplanes formicivorans* gen. nov., sp. nov., a novel sulfate-reducing bacterium isolated from a blackish meromictic lake, and emended description of the family *Desulfomicrobiaceae*. Int. J. Syst. Evol. Microbiol..

[75] Souza P.M., Goulart F.R.V., Marques J.M., Bizzo H.R., Blank A.F., Groposo C., Sousa M.P., Vólaro V., Alviano C.S., Moreno D.S.A. (2017). Growth inhibition of sulfate-reducing bacteria in produced water from the petroleum industry using essential oils. Molecules.

[76] Kuever J., Konneke M., Galushko A., Drzyzga O. (2001). Reclassification of *Desulfobacterium phenolicum* as *Desulfobacula phenolica* comb. nov. and description of strain Sax^T^ as *Desulfotignum balticum* gen. nov., sp. nov. Int. J. Syst. Evol. Bacteriol..

[77] Schink B., Thiemann V., Laue H., Friedrich M.W. (2002). *Desulfotignum phosphitoxidans* sp. nov., a new marine sulfate reducer that oxidizes phosphite to phosphate. Arch. Microbiol..

[78] Ommedal H., Torsvik T. (2007). *Desulfotignum toluenicum* sp. nov., a novel toluene-degrading, sulphate-reducing bacterium isolated from an oil-reservoir model column. Int. J. Syst. Evol. Microbiol..

[79] Li X.-X., Liu J.-F., Yao F., Wu W.-L., Yang S.-Z., Mbadinga S.M., Gu J.-D., Mu B.Z. (2016). Dominance of *Desulfotignum* in sulfate-reducing community in high sulfate production-water of high temperature and corrosive petroleum reservoirs. Int. Biodeterior. Biodegrad..

[80] Belyakova E.V., Rozanova E.P., Borzenkov I.A., Tourova T.P., Pusheva M.A., Lysenko A.M., Kolganova T.V. (2006). The new facultatively chemolithoautotrophic, moderately halophilic, sulfate-reducing bacterium *Desulfovermiculus halophilus* gen. nov., sp. nov., isolated from an oil field. Microbiology.

[81] Davey M.E., Wood W.A., Key R., Nakamura K., Stahl D.A. (1993). Isolation of three species of *Geotoga* and *Petrotoga*: Two new genera, representing a new lineage in the bacterial line of descent distantly related to the “*Thermotogales*”. Syst. Appl. Microbiol..

[82] Liang R., Davidova I.A., Marks C.R., Stamps B.W., Harriman B.H., Stevenson B.S., Duncan K.E., Suflita J.M. (2016). Metabolic capability of a predominant *Halanaerobium* sp. in hydraulically fractured gas wells and its implication in pipeline corrosion. Front. Microbiol..

[83] Booker A.E., Borton M.A., Daly R.A., Welch S.A., Nicora C.D., Hoyt D.W., Wilson T., Purvine S.O., Wolfe R.A., Sharma S. (2017). Sulfide generation by dominant *Halanaerobium* microorganisms in hydraulically fractured shales. mSphere.

[84] Semenova E.M., Grouzdev D.S., Tourova T.P., Nazina T.N. (2019). Physiology and genomic characteristics of *Geotoga petraea*, a bacterium isolated from a low-temperature oil reservoir (Russia). Microbiology.

[85] Oren A., Whitman W.B. (2015). “Halanaerobium” in Bergey’s Manual of Systematics of Archaea and Bacteria.

[86] Davis J.P., Struchtemeyer C.G., Elshahed M.S. (2012). Bacterial communities associated with production facilities of two newly drilled thermogenic natural gas wells in the Barnett Shale (Texas, USA). Microb. Ecol..

[87] Murali Mohan A., Hartsock A., Bibby K.J., Hammack R.W., Vidic R.D., Gregory K.B. (2013). Microbial community changes in hydraulic fracturing fluids and produced water from shale gas extraction. Environ. Sci. Technol..

[88] Cluff M.A., Hartsock A., Macrae J.D., Carter K., Mouser P.J. (2014). Temporal changes in microbial ecology and geochemistry in produced water from hydraulically fractured marcellus shale gas wells. Environ. Sci. Technol..

[89] Bhupathiraju V.K., Oren A., Sharma P.K., Tanner R.S., Woese C.R., McInerney M.J. (1994). *Haloanaerobium salsugo* sp. nov., a moderately halophilic, anaerobic bacterium from a subterranean brine. Int. J. Syst. Bacteriol..

[90] Bhupathiraju V.K., McInerney M.J., Woese C.R., Tanner R.S. (1999). *Haloanaerobium kushneri* sp. nov., an obligately halophilic, anaerobic bacterium from an oil brine. Int. J. Syst. Bacteriol..

[91] Ravot G., Magot M., Ollivier B., Patel B.K., Ageron E., Grimont P.A., Thomas P., Garcia J.L. (1997). *Haloanaerobium congolense* sp. nov., an anaerobic, moderately halophilic, thiosulfate- and sulfur-reducing bacterium from an African oil field. FEMS Microbiol. Lett..

[92] Tourova T.P., Sokolova D.S., Semenova E.M., Ershov A.P., Grouzdev D.S., Nazina T.N. (2022). Genomic and physiological characterization of halophilic bacteria of the genera *Halomonas* and *Marinobacter* from petroleum reservoirs. Microbiology.

[93] Yang N., Ding R., Liu J. (2022). Synthesizing glycine betaine via choline oxidation pathway as an osmoprotectant strategy in Haloferacales. Gene.

[94] Obrazstova A.Y., Shipin O.V., Bezrukova L.V., Belyaev S.S. (1988). Properties of the coccoid methylotrophic methanogen. Microbiology.

[95] Christman G.D., León-Zayas R.I., Summers Z.M., Biddle J.F. (2020). Methanogens within a high salinity oil reservoir from the Gulf of Mexico. Front. Microbiol..

[96] Katayama T., Yoshioka H., Mochimaru H., Meng X.-Y., Muramoto Y., Usami J., Ikeda H., Kamagata Y., Sakata S. (2014). *Methanohalophilus levihalophilus* sp. nov., a slightly halophilic, methylotrophic methanogen isolated from natural gas-bearing deep aquifers, and emended description of the genus *Methanohalophilus*. Int. J. Syst. Evol. Microbiol..

[97] Wilharm T., Zhilina T.N., Hummel P. (1991). DNA-DNA hybridization of methylotrophic halophilic methanogenic bacteria and transfer of *Methanococcus halophilus* VP to the genus *Methanohalophilus* as *Methanohalophilus halophilus* comb. nov. Int. J. Syst. Bacteriol..

[98] L’Haridon S., Haroun H., Corre E., Roussel E., Chalopin M., Pignet P., Balière C., la Cono V., Jebbar M., Yakimov M. (2020). *Methanohalophilus profundi* sp. nov., a methylotrophic halophilic piezophilic methanogen isolated from a deep hypersaline anoxic basin. Syst. Appl. Microbiol..

[99] Guan Y., Ngugi D.K., Vinu M., Blom J., Alam I., Guillot S., Ferry J.G., Stingl U. (2019). Comparative genomics of the genus *Methanohalophilus*, including a newly isolated strain from Kebrit Deep in the Red Sea. Front. Microbiol..

