# Supplementary material for: Metagenomic and Culture-Based Analyses of Microbial Communities from Petroleum Reservoirs with High-Salinity Formation Water, and Their Biotechnological Potential
Source: Biology (Basel). 2023 Oct 2;12(10):1300. doi: 10.3390/biology12101300 (PMC10604348; doi:10.3390/biology12101300)
Supplement: Supplementary file 1 [file biology-12-01300-s001.zip › SUPPLEMENTARY MATERIALS.pdf]

biology MDPI

Supplementary Materials

Metagenomic and culture-based analyses of microbial communities from petroleum reservoirs with high-salinity formation water, and their biotechnological potential

Vitaly V. Kadnikov[1], Nikolai V. Ravin[1], Diyana S. Sokolova[2], Ekaterina M. Semenova[2], Salimat K. Bidzhieva[2], Alexey V. Beletsky[1], Alexey P. Ershov[2], Tamara L. Babich[2], Marat R. Khisametdinov[3], Andrey V. Mardanov[1] and Tamara N. Nazina[2]*

[1] Institute of Bioengineering, Research Center of Biotechnology of the Russian Academy of Sciences, Moscow, Russia,
[2] Winogradsky Institute of Microbiology, Research Center of Biotechnology of the Russian Academy of Sciences, Moscow, Russia,
[3] Tatar Scientific Research and Design Institute of Oil "Tatneft", Bugulma 423236, Russia
*Correspondence: Tamara N. Nazina (nazina@inmi.ru)

This file includes:

Figures S1 to S6

Tables S1, S2, S6, and S7

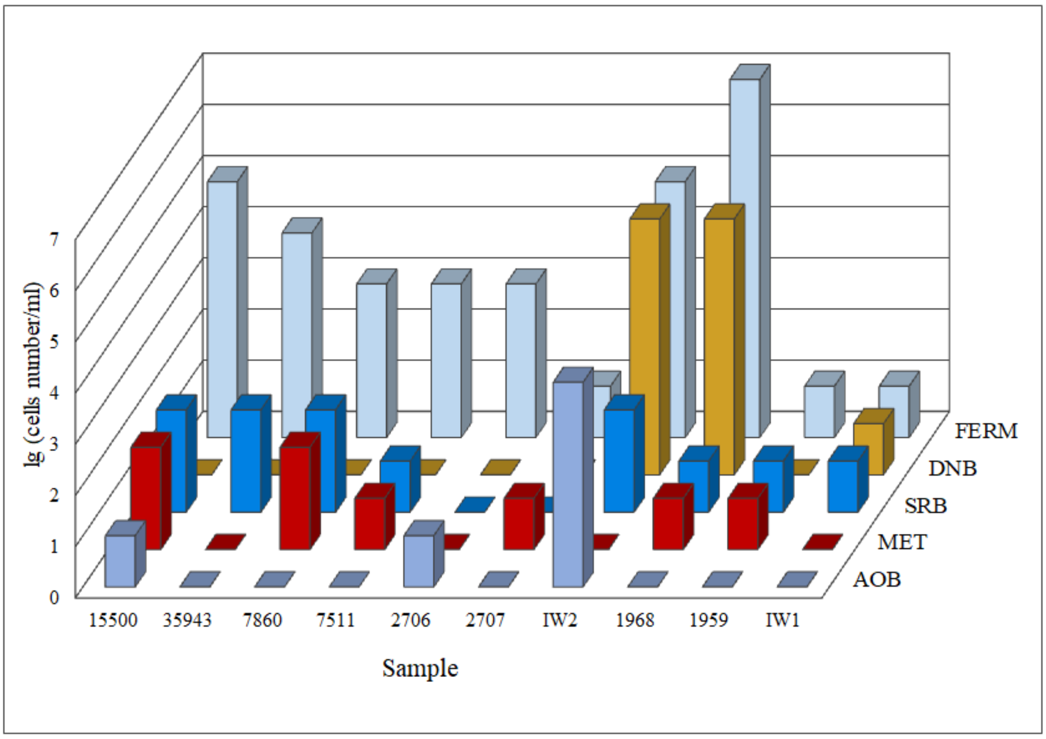

Figure S1. Numbers of aerobic and anaerobic microorganisms in injection and production water samples from oil fields located in Tatarstan (Russia). Designations: AOB, aerobic organotrophic bacteria; FERM, fermentative bacteria; DNB, denitrifying bacteria; SRB, sulfate-reducing bacteria; MET, methanogenic archaea. Samples of injection water are designated as IW1 and IW2, the rest are water samples from production wells.

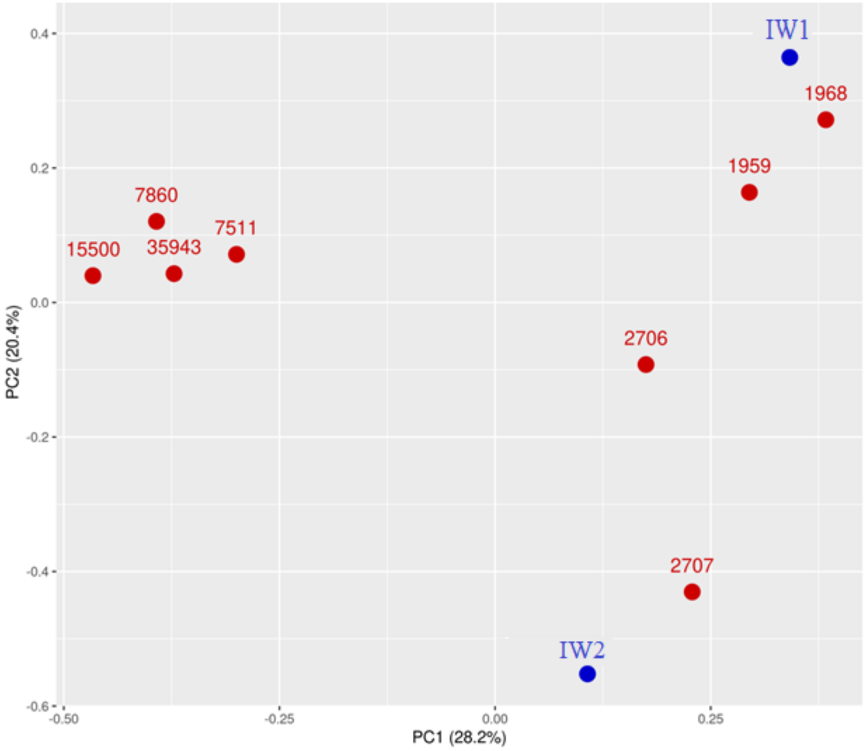

Figure S2. Principle components analysis (PCA) of the composition of microbial communities of injection and production water from oil fields of Tatarstan, based on the relative abundance of operational taxonomic units (≥97% similarity of 16S pPHK gene sequences) of prokaryotes.

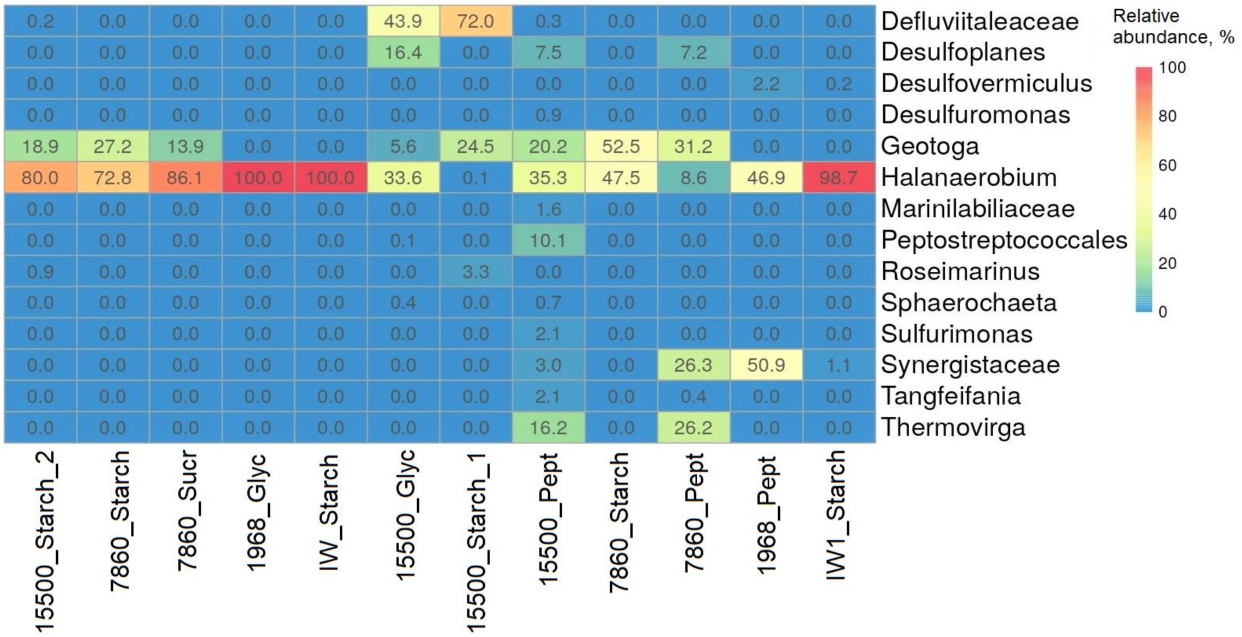

Figure S3. Heatmap of the relative abundance of bacteria at the genus level based on 16S rRNA amplicon sequencing (SILVA Database) in fermentative enrichments obtained from the production and injection water samples. Representation of the genus was calculated as sequence proportions divided by total sequence count in each library. Columns are clustered using correlation distance and average linkage. Enrichments were obtained in media with starch, peptone (Pept), sucrose (Sucr), and glycerol (Glyc).

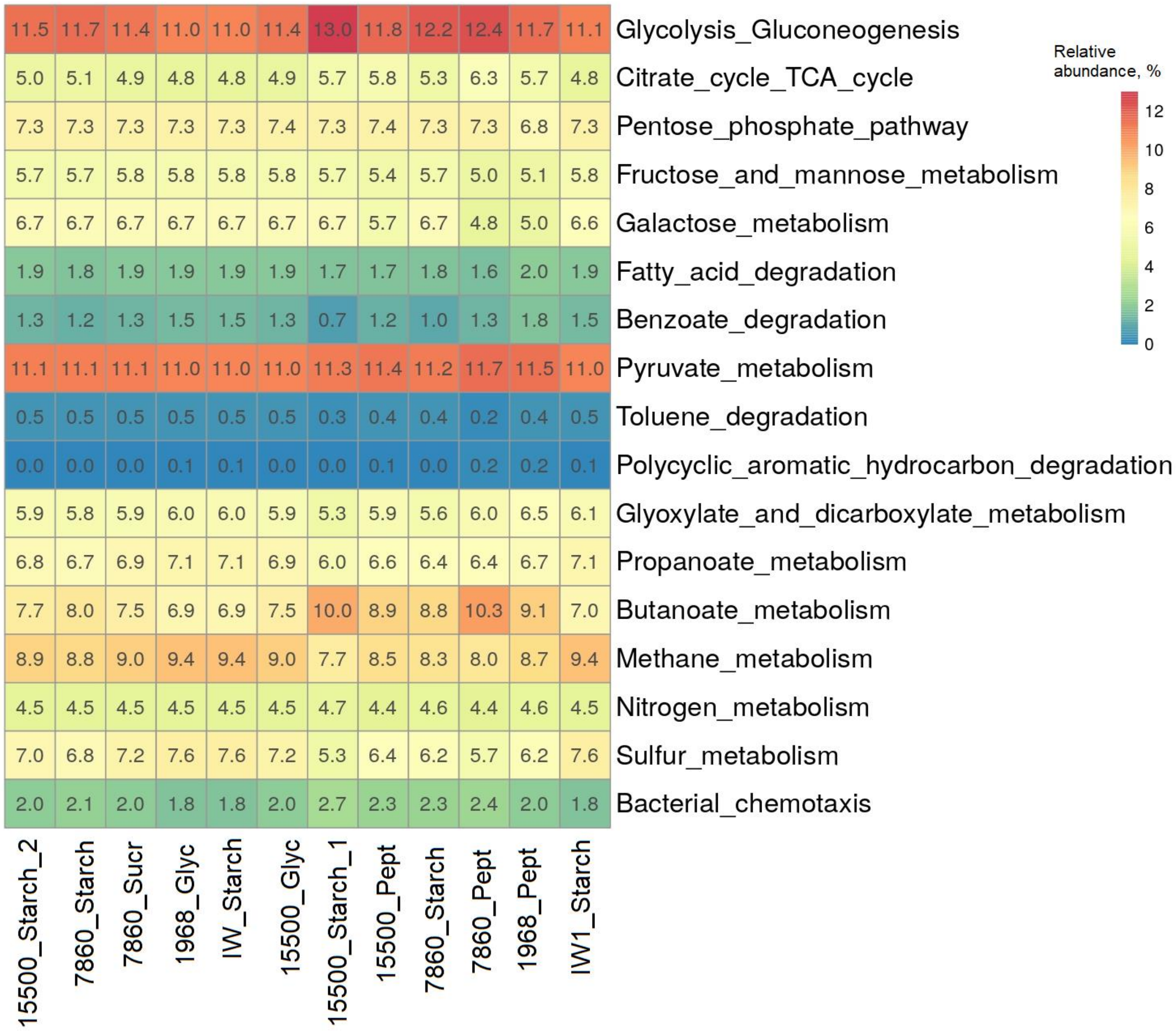

Figure S4. Heatmap showing metabolic pathways (KEGG Database) in fermentative enrichments obtained from the production and injection water samples.

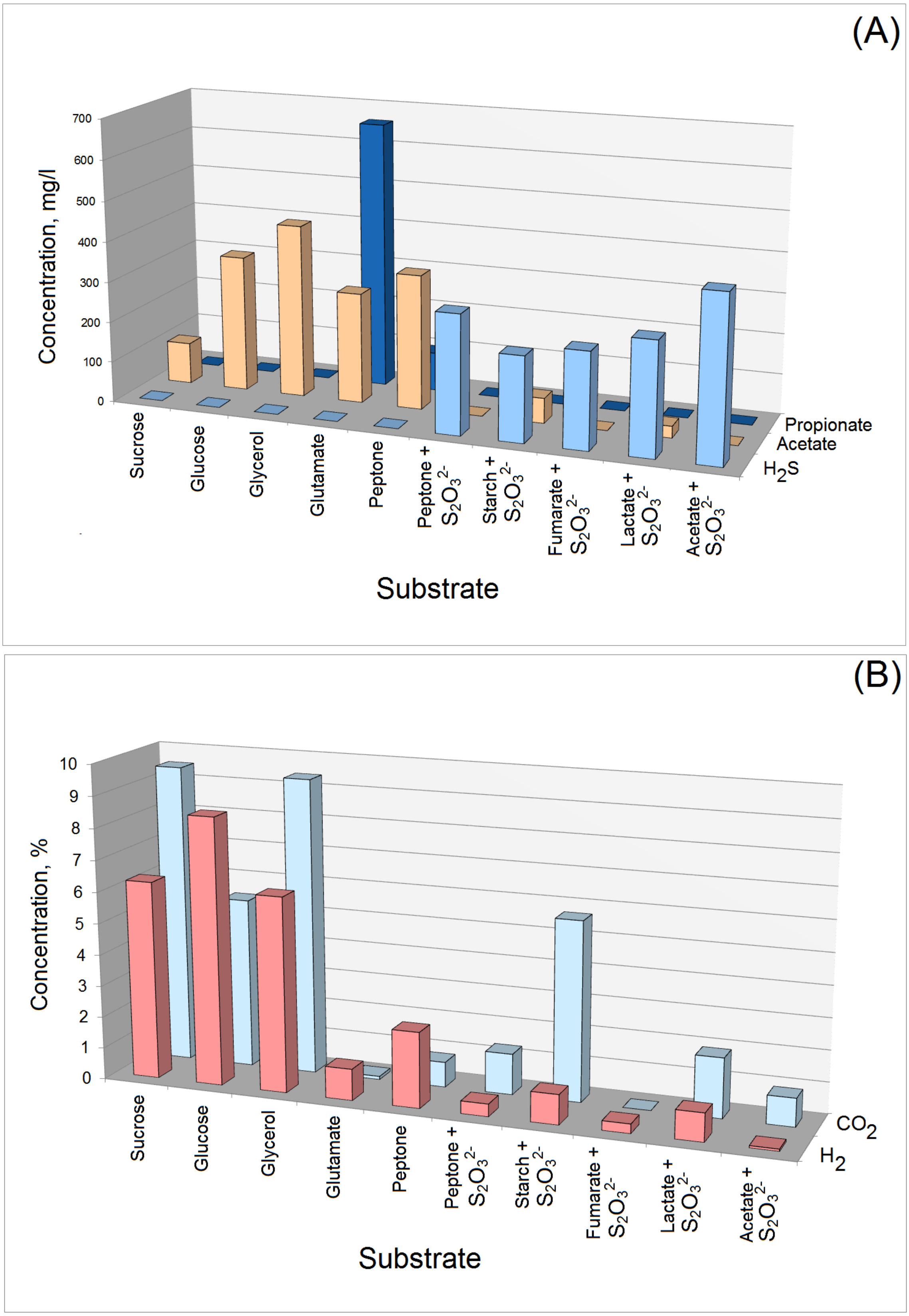

Figure S5. Concentrations of low fatty acids and sulfide in a medium (A) and CO_2 and H_2 in a gas phase (%, v/v; B) produced by PW15500 fermentative enrichment in media with various organic substrates amended with sodium thiosulfate or without it after 14 days of incubation at 37 °C.

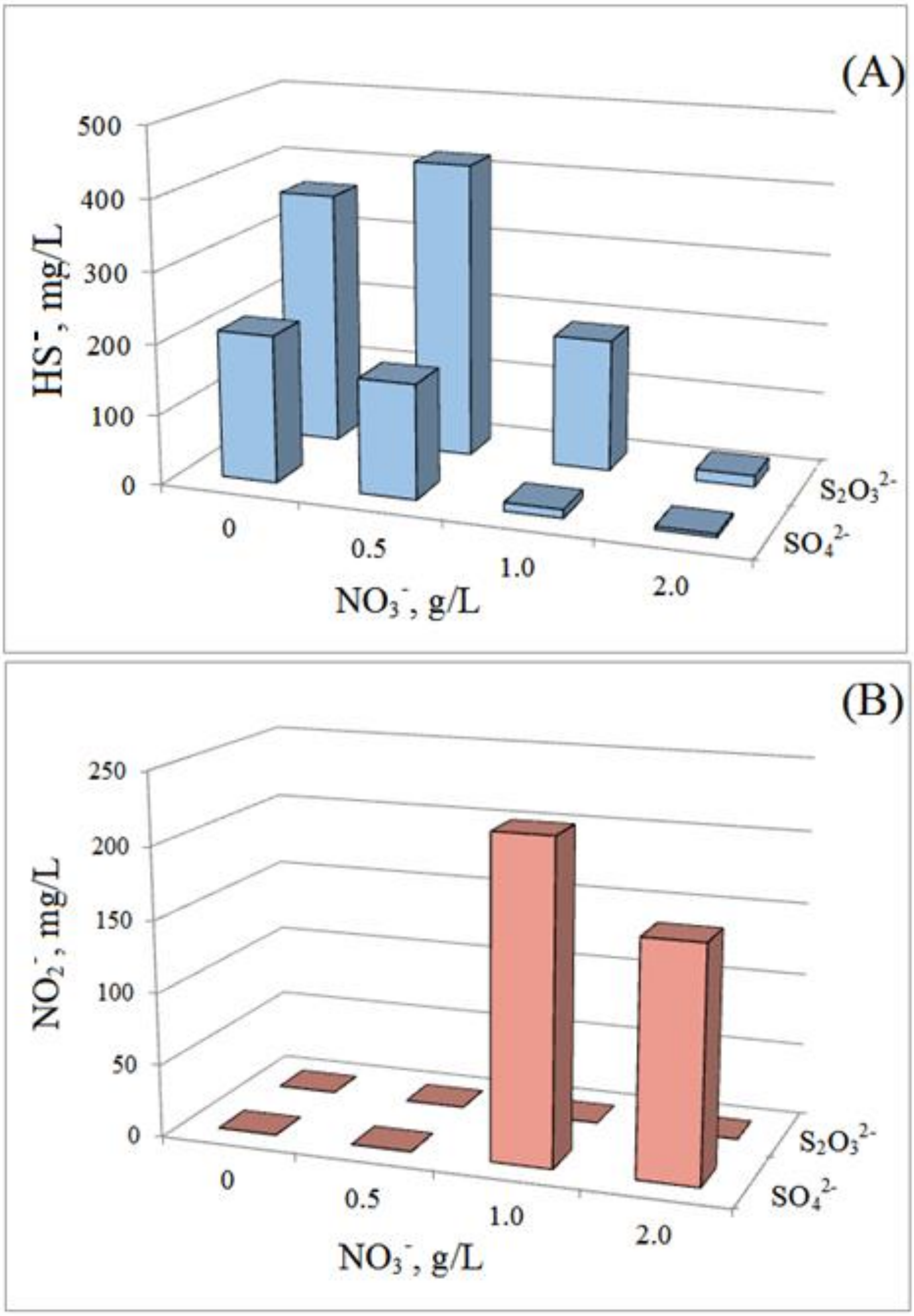

Figure S6. Production of sulfide (A) and nitrite (B) by PW15500_Pept fermentative enrichment in media with peptone and thiosulfate or sulfate and different initial nitrate concentrations. Enrichments were incubated at 22 °C for 28 days.

Table S1. Characteristics of the oil reservoirs

**Parameter**	**Romashkinskoe oilfield, bed 302**	**Arkhangelskoe oilfield**	**Novo-Elhovskoe oilfield**	**Sabanchinskoe oilfield**
Samples from wells	PW15500, PW35943	PW7860, IW2	PW7511*, PW2706, PW2707	IW1, PW1968, PW1959
Horizon	Bashkirian	Bashkirian	Pashiysky	Bobrikovsky
Type of collector, lithology	Carbonate	Limestone organogenic with sandstone and dolomites	Terrigenous, sandstone	Terrigenous, sandstone
Average depth below sea level, m	875	830, 988–998	1225–1745	1586
Average porosity, %	12.4	12.8	24.0	20.5
Average permeability, μm^2	0.086	0.278	0.479	0.176
Effective oil-saturated reservoir capacity, m	5.0	3.2–12.8	8.8	2.4
The formation temperature, °C	19–23	20	20.2–21.3	23.3
Oil density in surface condition, g/cm^3 (20 °C)	0.908	0.924	0.862	0.856
Average oil viscosity in reservoir condition, mPa·s	43.6	23.2	4.1	58.0
Water cut (average), %	82–85	68–93	61–96	33

*7511, Bobrikovsky horizon, the formation temperature is 25–27 °C.

Table S2. Number of sequences and diversity indices in the libraries of the 16S rRNA gene fragments of microorganisms in injection and production water samples from oil fields located in Tatarstan

**Sample**	**PW15500**	**PW35943**	**PW7860**	**PW7511**	**PW2706**	**PW2707**	**PW1968**	**PW1959**	**IW1**	**IW2**
Number of reads	9268	8372	10113	7184	5741	8985	7390	7958	3756	8020
OTUs number	249	171	99	122	71	383	51	120	47	410
CHAO	189.7	137.7	79.1	113.9	68.9	312.7	43.1	98.9	49.8	359.2
Shannon index (H10)	0.932	1.17	0.534	1.11	1.21	1.56	0.678	1.09	0.507	1.88

Table S6. Taxonomic affiliation of pure cultures isolated from production and injection water samples

Strain	GenBank number	Closest type strain according to 16S rRNA gene, acc. no.	16S rRNA gene similarity, %	Isolation source, well no.	Substrate, acceptor of electrons, NaCl content used for strain isolation	Temperature of incubation, °C
T1S	OP503475	*Bacillus licheniformis* DSM 13, KX785171.1	100.0	PW15500	Sucrose, O_2, 5% NaCl	37
T21-1F	OP588951		100.0	PW15500	Fumarate, O_2, 4.5% NaCl	28
T14KS	OP849624	*Cytobacillus oceanisediminis* strain H2, OL875278.1	100.0	IW1	Starch, NO_3^-, 10% NaCl	37
T21-11F			100.0	PW1968	Fumarate, O_2, 4.5% NaCl	28
9S37	OP459266	*Mesobacillus jeotgali* strain DSM 18226, MZ540442.1	99.1	PW7860	Sucrose, 2.4% NaCl	37
T9KN	OP849652	*Mesobacillus jeotgali* strain YKJ-10, NR_025060.1	100.0	PW7860	Starch, NO_3^-, 7% NaCl	37
T1KP	OP849684		99.9	PW15500	Starch, NO_3^-, 5% NaCl	37
9S	OP459272	*Geotoga subterranea* strain CC-1, NR_029145.2	99.7	PW15500	Peptone, $S_2O_3^{2-}$, 5% NaCl	37
11S	OP459302		99.7	PW15500	Peptone, $S_2O_3^{2-}$, 5% NaCl	37
T1GS	OP503477		99.8	PW15500	Glycerin, S^0, 5% NaCl	25
9g48	OP459271		99.7	PW7860	Glucose, 2.4% NaCl	48
9f37	OP459263	*Halanaerobium acetethylicum* strain EIGI, NR_118801.1	99.2	PW7860	Fumarate, 10% NaCl	37
T11P	OP503444	*Halanaerobium congolense* strain SEBR 4224, NR_026044.1	99.8	PW1968	Glycerin + peptone, 10% NaCl	37
1KS37	OP459260	*Halanaerobium praevalens* strain GSL, NR_074859.1	99.8	PW15500	Starch, 10% NaCl	37
T9K	OP459305	*Halanaerobium saccharolyticum* subsp. *senegalense* strain DSM 7379, NR_026258.1	98.0	PW7860	Starch, 10% NaCl	37
1S37	OP503479	*Sphaerochaeta associata* strain GLS2, KY643672.1	97.4	PW15500	Sucrose, 2.4% NaCl	37
1S48	OP459261		97.1	PW15500	Sucrose, 2.4% NaCl	48
4SSU	OP848502		98.0	PW15500	Sucrose, $S_2O_3^{2-}$, 1% NaCl	37
PS50	OQ348127	*Desulfoplanes formicivorans* strain Pf12B, NR_135886.1	98.6	PW7860	Formate, $S_2O_3^{2-}$, 5% NaCl	22
T1FT	OQ348135	*Tangfeifania diversioriginum* strain G22, NR_134211.1	98.5	PW15500	Pyruvate, S^0, 5% NaCl	22

Table S7. Surface tension (ST) and interfacial* tension (IT) of culture liquid with bacteria grown on various substrates in aerobic conditions

Strain	Genus, species, growth conditions	Substrate	ST, mN/m	IT, mN/m
T21-1F	*Bacillus licheniformis*, aerobic	Yeast extract	52.6	26.1
		Glycerol	65.4	26.8
		Starch	55.0	20.6
		Molasses	56.2	**5.3**
		Peptone	51.3	**9.5**
		Pyruvate	54.8	28.0
		Sucrose	43.3	18.2
9S37	*Mesobacillus jeotgali*, aerobic	Yeast extract	72.0	41.1
		Peptone	68.0	40.1
T21-11F	*Cytobacillus oceanisediminis*, aerobic	Yeast extract	70.0	26.6
		Glycerol	63.3	28.3
		Starch	58.4	22.2
		Molasses	62.4	29.0
		Peptone	67.0	28.3
		Pyruvate	73.4	30.2
		Sucrose	52.3	31.3
1S37	*Sphaerochaeta* sp., anaerobic	Yeast extract	59.9	38.1
		Molasses	61.3	33.9
		Peptone	56.8	28.8
		Sucrose	62.0	32.2
1S48	*Sphaerochaeta* sp., anaerobic	Yeast extract	55.9	45.1
		Molasses	56.8	33.0
		Peptone	59.9	36.3
		Sucrose	47.4	17.6
9f37	*Halanaerobium acetethylicum*, anaerobic	Sucrose	67.1	33.6
9g48	*Geotoga subterranea*, anaerobic	Sucrose	62.4	41.2

* The interfacial tension was measured against hexadecane at a temperature of 22 °C.
